# Cell-Type-Specific TEV Protease Cleavage Reveals Cohesin Functions in *Drosophila* Neurons

**DOI:** 10.1016/j.devcel.2007.12.009

**Published:** 2008-02-12

**Authors:** Andrea Pauli, Friederike Althoff, Raquel A. Oliveira, Stefan Heidmann, Oren Schuldiner, Christian F. Lehner, Barry J. Dickson, Kim Nasmyth

**Affiliations:** 1 Department of Biochemistry, University of Oxford, Oxford OX1 3QU, UK; 2 Institute of Zoology, University of Zurich, 8057 Zurich, Switzerland; 3 Department of Genetics, University of Bayreuth, 95440 Bayreuth, Germany; 4 Stanford University, Department of Biological Sciences, Stanford, CA 94305, USA; 5 Institute of Molecular Pathology, 1030 Vienna, Austria

**Keywords:** DEVBIO, CELLCYCLE, MOLNEURO

## Abstract

Cohesin is a highly conserved multisubunit complex that holds sister chromatids together in mitotic cells. At the metaphase to anaphase transition, proteolytic cleavage of the α kleisin subunit (Rad21) by separase causes cohesin's dissociation from chromosomes and triggers sister-chromatid disjunction. To investigate cohesin's function in postmitotic cells, where it is widely expressed, we have created fruit flies whose Rad21 can be cleaved by TEV protease. Cleavage causes precocious separation of sister chromatids and massive chromosome missegregation in proliferating cells, but not disaggregation of polytene chromosomes in salivary glands. Crucially, cleavage in postmitotic neurons is lethal. In mushroom-body neurons, it causes defects in axon pruning, whereas in cholinergic neurons it causes highly abnormal larval locomotion. These data demonstrate essential roles for cohesin in nondividing cells and also introduce a powerful tool by which to investigate protein function in metazoa.

## Introduction

The investigation of nonmitotic functions of proteins essential for cell proliferation poses a major technical challenge: namely, how to inactivate such proteins without compromising cell proliferation. A good example is the highly conserved multisubunit complex called cohesin (Guacci et al., 1997; Michaelis et al., 1997), which holds the products of DNA replication (sister chromatids) together and thereby ensures their segregation to opposite poles of the cells during mitosis and meiosis (reviewed in Nasmyth and Haering, 2005 and Hirano, 2006). Cohesin forms a large tripartite ring composed of a pair of Structural Maintenance of Chromosome (SMC) proteins, SMC1 and SMC3, and an α kleisin protein, Scc1/Rad21, whose cleavage by separase causes cohesin's dissociation from chromosomes and triggers sister-chromatid disjunction at the metaphase to anaphase transition. Sister-chromatid cohesion requires two other non-SMC subunits, namely, Pds5 and Scc3/SA, that bind to cohesin's α kleisin subunit. The establishment of cohesion depends on the cohesin loading complex Scc2/Scc4 and on the acetyl-transferase Eco1/Ctf7.

The fact that cohesin forms a ring whose opening releases it from chromatin has led to the suggestion that it holds sister DNAs together by using a topological mechanism (Gruber et al., 2003). Importantly, this type of function could also be of value in regulating aspects of chromosome organization that are independent of sister-chromatid cohesion and are not directly required for chromosome segregation. It is notable in this regard that the majority of cohesin is removed from chromosome arms during prophase/prometaphase in most eukaryotic cells by a separase-independent mechanism (Gandhi et al., 2006; Kueng et al., 2006). Only cohesin that subsequently persists on chromosomes is cleaved by separase at the onset of anaphase (Waizenegger et al., 2000). As a consequence, there exists a large pool of cohesin ready to reassociate with chromosomes as soon as cells exit from mitosis during telophase. Cohesin is therefore tightly associated with chromosomes for much of the cell-division cycle and could have important functions on unreplicated genomes.

Much evidence has emerged recently that cohesin might have important roles in regulating gene expression (reviewed in Dorsett, 2007). Approximately half of the cases of a multisystem developmental disorder in humans called Cornelia de Lange syndrome (CdLS), which is characterized by mental retardation, upper limb abnormalities, growth delay, and facial dysmorphisms, are caused by mutations in genes encoding NIPBL/Delangin (the human Scc2 ortholog), SMC1A, or SMC3 (Deardorff et al., 2007; Krantz et al., 2004; Musio et al., 2006; Tonkin et al., 2004). Because even severe cases of CdLS appear not to be accompanied by defects in sister-chromatid cohesion, it has been suggested that CdLS is caused by misregulated gene expression during embryonic development. Consistent with this possibility, the *Drosophila* Scc2 ortholog, Nipped-B, facilitates long-range enhancer-promotor interactions, at least for certain genes whose regulatory sequences have been mutated (Dorsett et al., 2005; Rollins et al., 1999). Furthermore, mutations in *mau-2*, the *Caenorhabditis elegans Scc4* ortholog, cause defects in axon guidance (Bernard et al., 2006; Takagi et al., 1997). Recently, two cohesin subunits, Scc1/Rad21 and SMC3, have been implicated in expression of the hematopoietic transcription factors *runx1* and *runx3* in zebrafish (Horsfield et al., 2007).

Despite these findings, it cannot be excluded that developmental “cohesinopathies” are in fact caused by “knock on” effects of compromising the establishment or maintenance of sister-chromatid cohesion. In the case of CdLS, for example, haploinsufficiency of NIPBL/Delangin might cause cell-type-specific sister-chromatid cohesion defects (Kaur et al., 2005) that would be overlooked by examining this process in only one type of cell. It is therefore vital to develop methods that permit observation of the effects on gene expression and development of eliminating cohesin's function completely without interfering with cell proliferation.

To analyze cohesin's function in a more sophisticated manner than hitherto possible, to our knowledge, in metazoa, we have used the tobacco etch mosaic virus (TEV) protease to cleave cohesin's α kleisin subunit in *Drosophila melanogaster* in a cell-type-specific and/or temporally controlled manner. This process opens the cohesin ring and presumably abolishes its topological embrace of chromatin fibers (Gruber et al., 2003). As expected, expression of TEV protease in proliferating cells of fly embryos whose sole form of Rad21 contains TEV-cleavage sites causes precocious separation of sister chromatids and has a devastating effect on chromosome segregation. More remarkably, TEV-induced Rad21 cleavage in postmitotic neurons is lethal. It causes defects in the developmental axon pruning of mushroom-body γ neurons within pupal brains and defects in cholinergic neurons that result in highly abnormal larval locomotion.

## Results

### A System to Inactivate Pre-Existing Cohesin Complexes

**Figure 1:**
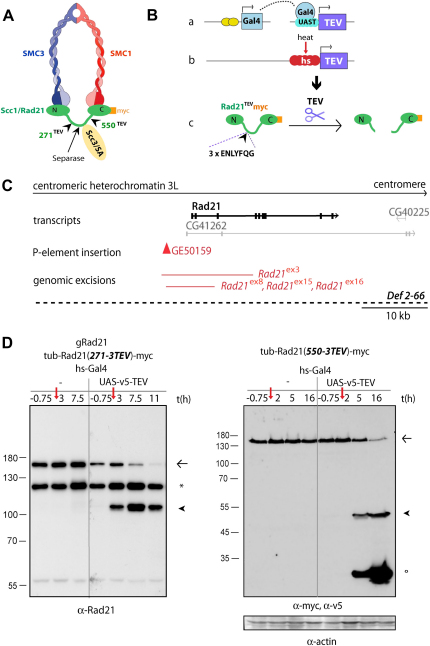
Outline of the TEV-Cleavage System (A) Schematic of the cohesin complex containing TEV-cleavable Rad21 (green), SMC1 (red), SMC3 (blue), and Scc3/SA (yellow). Cleavage of Rad21 by separase occurs in the flexible linker region. Arrowheads indicate the sites of insertion of TEV-recognition sequences (numbers refer to amino acid positions). (B) Outline of the TEV-cleavage system showing two alternative methods to express TEV in vivo in flies. (a) UAS-TEV is controlled by the UAS/GAL4 system, enabling TEV expression by specific Gal4 driver lines. (b) TEV directly fused to the heat-shock promotor allows for its ubiquitous induction in a time-specific manner. (c) Once expressed, catalytically active TEV protease cleaves Rad21^TEV^. (C) Representation of the genomic region of the *Rad21* locus. The *Rad21* gene (CG17436) resides in the centric heterochromatin of chromosome 3L. The exon-intron structure of the *Rad21* mRNA is shown in bold. EST-based transcript predictions of neighboring genes are depicted in lighter gray. The EP element GE50159 4 kb upstream of the transcriptional start of *Rad21* is represented by a red triangle. The four independently generated imprecise excision mutants of *Rad21* lack the chromosomal intervals indicated by solid, red lines. The *Rad21* locus is missing in the γ-ray-induced deficiency *Def 2-66* (dashed line). The scale bar is 10 kb. (D) Pupal protein extracts were prepared before (t = −0.75 hr) and at different time points after a 45 min heat shock at 37°C (red arrow). Western blot analysis with antibodies against endogenous Rad21 (left panel) or myc (right panel) shows full-length Rad21^TEV^ (arrow) and the C-terminal TEV-cleavage product (arrowhead) as well as gRad21 (asterisk). V5-tagged TEV protease is detected by probing with v5 antibodies (open circle). Actin was used as a loading control. A molecular weight marker (in kDa) is shown on the left.

To inactivate cohesin, we chose cleavage of its α kleisin subunit (Rad21). Although this does not directly affect any known functional domain of Rad21, it severs and thereby opens cohesin's tripartite ring (Figure 1A), leading to its rapid dissociation from chromosomes. To do this in *Drosophila*, it was necessary first to create a *Rad21* mutant strain, second to complement the *Rad21* mutation with a version of *Rad21* that contains cleavage sites for a site-specific protease, and lastly to express a version of the protease that can accumulate within nuclei in a tissue-specific and/or time-dependent manner. We used TEV protease because it has been used successfully for this purpose in the budding yeast *Saccharomyces cerevisiae* (Uhlmann et al., 2000).

### Generation of a *Rad21* Mutant Fly

The *Rad21* gene (CG17436) is located within the centric heterochromatin of chromosome 3L (Markov et al., 2003), but no mutants were available. To create *Rad21* mutations, a P element inserted 4 kb upstream of the transcriptional start of *Rad21* was remobilized by P element Transposase. Among the homozygous lethal stocks, we identified four independent *Rad21* deletion alleles by using PCR (*Rad21^ex3^*, *Rad21^ex8^*, *Rad21^ex15^*, *Rad21^ex16^*) (Figure 1C). All four alleles lack exons 1 and 2, which encode the highly conserved N terminus of Rad21 that interacts with the ATPase head of SMC3 (Figure 1C; Figure S1, see the Supplemental Data available with this article online) (Haering et al., 2002).

Homozygous mutant *Rad21* embryos develop normally during early embryogenesis (data not shown). DNA staining suggests that mitoses are normal throughout the first 16 epidermal cell divisions. Late mitoses and cell divisions in embryonic neural precursors also appear to be unaffected (data not shown). The maternal gene product is presumably sufficient to execute the embryonic cell-division program. Despite this, most (95%) homozygous mutant embryos die before hatching. The rare mutant larvae that hatch possess almost no motor activity and fail to grow. It is therefore conceivable that embryonic death arises from a defective nervous system.

### Flies Expressing TEV-Cleavable Rad21 Are Viable and Fertile

To rescue *Rad21* mutants, we generated transgenic flies that express C-terminally myc-epitope-tagged versions of Rad21 with TEV-cleavage sites. A tandem array of three TEV consensus recognition sequences was inserted into four poorly conserved and putatively unstructured regions within Rad21's central domain (Figure 1A; for details, see Figure S1). The cleavability of these proteins was initially tested by cotransfecting tissue-culture cells with vectors expressing TEV-cleavable Rad21 (Rad21^TEV^) and TEV protease. This showed that all four versions of Rad21^TEV^ were efficiently cleaved (data not shown). Equally important, Rad21^TEV^ with three TEV sites at position 271 or 550 as well as a version lacking TEV insertions restored full viability and fertility of homozygous *Rad21^ex^* alleles when expressed from a tubulin promotor (Table S2). We were thus able to generate fly stocks that carry Rad21^TEV^ as their sole source of Rad21.

### Efficient TEV-Induced Rad21 Cleavage In Vivo

To test whether flies can tolerate TEV protease, we created transgenic flies that express v5-epitope-tagged TEV in an inducible manner, either directly from the heat-shock promotor (hs-TEV) or under the control of the Gal4/UAS system (Brand and Perrimon, 1993) (Figures 1Ba and 1Bb). TEV tagged with three nuclear localization sequences (NLS) accumulated within nuclei and did not cause any notable phenotypes when expressed ubiquitously or in a tissue-specific manner by using a variety of different Gal4 driver lines (data not shown). Western blot showed that TEV induction caused the appearance of cleavage fragments of the expected size from Rad21^TEV^ proteins, but not from endogenous Rad21 or transgenic Rad21 proteins (Figure 1D and data not shown). Heat shock led to the accumulation of TEV and Rad21-cleavage fragments more rapidly when the protease was expressed from hs-TEV compared to hs-Gal4/UAS-TEV (data not shown). Importantly, TEV induction led to cleavage of most of the Rad21^TEV^ pool within a few hours.

### TEV-Induced Rad21 Cleavage Causes Chromosome Missegregation

To investigate the consequences of Rad21 cleavage in a single cell cycle, we made use of the fact that zygotic expression is specifically switched on during embryonic cycle 14. Maternal Gal4 (α4-tub-Gal4) was used to drive expression of paternally contributed UAS-TEV in embryos containing Rad21^TEV^ as their sole source of Rad21. Western blot confirmed that the expression of TEV causes a reduction in the level of intact Rad21^TEV^ and the appearance of a ∼90 kDa TEV-cleavage fragment before mitosis 14 (Figure S2A). The residual full-length protein presumably stems from embryos (50%) that did not inherit the TEV protease-containing chromosome. These results suggest that most, if not all, Rad21^TEV^ is cleaved during cycle 14.

**Figure 2:**
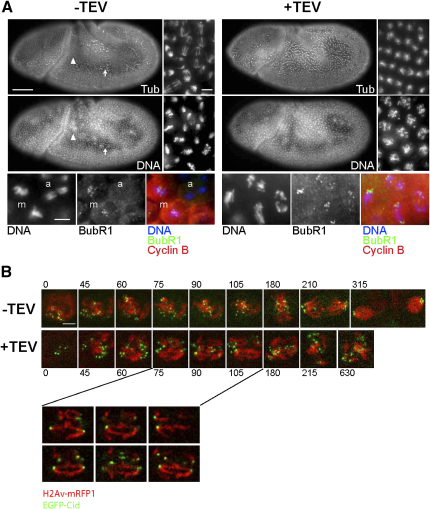
Cleavage of Rad21^TEV^ during Cycle 14 Causes Precocious Sister-Chromatid Separation and Transient Mitotic Arrest (A) Cycle 14 embryos that survived on Rad21^TEV^ and expressed maternally contributed Gal4 were fixed and double labeled (top rows) with anti-α-tubulin (Tub) and a DNA stain (DNA) or were triple labeled (bottom row) with DNA stain (blue), anti-BubR1 (green), and anti-Cyclin B (red). +TEV indicates the additional presence of the UAS-TEV transgene. The scale bars are 50 μm in the top left panel, 10 μm in the top right panel, and 10 μm in the bottom panel. (Top) Most cells in −TEV embryos have already completed mitosis 14 (arrowhead in whole embryo views). Dividing cells (arrow) during various mitotic stages (pro-, meta-, ana-, telophase) are shown in the high-magnification view. In +TEV embryos, the entire dorsolateral epidermis is arrested in mitosis. (Bottom) In −TEV embryos, high levels of BubR1 and Cyclin B are only observed during metaphase (m), whereas anaphase (a) cells do not stain for BubR1 and Cyclin B. Arrested cells of +TEV embryos are Cyclin B positive and have high levels of BubR1 on separated sister kinetochores. (B) Embryos surviving on Rad21^TEV^ and expressing either only maternal Gal4 (−TEV) or maternal Gal4-driven TEV protease (+TEV) were used for time-lapse imaging. DNA is marked with H2Av-mRFP1; kinetochores are marked with EGFP-Cid. The onset of chromosome condensation was set to zero. Time points are indicated in seconds. Whereas the top two rows represent Z projections, the bottom rows show single confocal sections. The scale bars are 2 μm. (−TEV) Chromosomes congress into a metaphase plate (t = 180), followed by anaphase (t = 210) and telophase (t = 315). (+TEV) Chromosomes fail to congress into a metaphase plate, and sister chromatids separate prematurely (t = 75–105). Note the substantial mitotic delay (t = 630).

Rad21^TEV^ cleavage had no effect on progression through the first 13 embryonic cell-division cycles, during which TEV is not expressed (data not shown). By contrast, as soon as zygotic expression is switched on, TEV had a devastating effect as cells embarked on mitosis 14. DNA staining and immunolabeling of embryos with anti-tubulin revealed the absence of any normal meta-, ana-, and telophase figures (Figure 2A). Despite the formation of bipolar spindles, condensed chromosomes failed to align on a metaphase plate and were found scattered throughout cells. Cells accumulated in this metaphase-like state, with high levels of Cyclin B and BubR1 concentrated at kinetochores.

These observations are consistent with the notion that Rad21 cleavage causes precocious loss of sister-chromatid cohesion. This would prevent the establishment of the tension at kinetochores needed to turn off the spindle-assembly checkpoint (SAC) and causes mitotic arrest (Logarinho et al., 2004; Tanaka, 2005). To test this, we used time-lapse microscopy to observe chromosomes marked with histone H2Av-mRFP1 and kinetochores marked with EGFP-Cid. This revealed that, upon Rad21 cleavage, chromosomes condense during prophase of cycle 14, usually with paired, presumably sister, kinetochores similar to those found in a wild-type strain (Figure 2B, t = 0–60 s, compare Movie S1 [WT] and Movie S2 [Rad21-depleted]). However, during prometaphase, soon after biorientation, sister chromatids disjoin prematurely and often segregate to opposite poles. This highly abnormal process is asynchronous, with different chromosomes splitting at different times. As a result, chromosomes do not congress to a metaphase plate (Figure 2B; Movies S2 and S3). Exit from mitosis is delayed and cells arrest in a highly abnormal mitotic state, during which individual chromatids often lose their attachment to spindle poles, sometimes reorient, and move between poles (Figure S2B). After ∼20 min, chromosome decondensation occurs abruptly and chromatids in the equatorial plane are cut by the cleavage furrow (Figure S2C; Movie S4). Although the mitotic arrest caused by Rad21 cleavage is only transient, mitosis nevertheless lasts approximately six times longer than in wild-type. These results are consistent with data from previous RNAi experiments in tissue-culture cells (Vass et al., 2003) and clearly show that Rad21 is essential for mitosis. We conclude that cohesin is necessary for sister-chromatid cohesion in the fly, as it is in yeast and vertebrate cells.

### Cohesin Binds to Defined Regions on Polytene Chromosomes

We next used TEV cleavage to address whether cohesin has a role in holding together the multiple DNA molecules of polytene chromosomes in salivary glands. These chromosomes are generated by repeated rounds of DNA replication without intervening mitoses (endoreduplication) (reviewed in Zhimulev et al., 2004).

**Figure 3:**
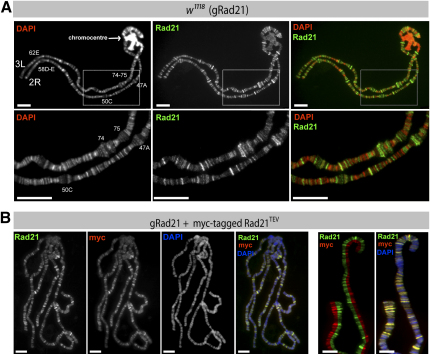
Cohesin Binds to Distinct Regions on Polytene Chromosomes (A) Polytene chromosomes of wild-type flies (*w^1118^*) were stained with Rad21 antibodies (green) and DAPI (DNA, red). The lower panel shows a higher-magnification view (2.5×). The strongly DAPI-stained heterochromatic chromocenter (arrow) is devoid of Rad21 staining. The scale bars are 20 μm. (B) Polytene chromosomes from flies expressing myc-tagged Rad21^TEV^ in addition to endogenous Rad21 were coimmunostained with antibodies against Rad21 (green) and myc (red). DNA was visualized with DAPI (blue). In the right two frames, part of one chromosome arm is shown at higher magnification with split Rad21- and myc channels. The scale bars are 20 μm in the left four frames and 10 μm in the right two frames.

Immunostaining of wild-type polytene-chromosome squashes showed that Rad21, detected with a Rad21-specific antibody, localizes mainly to interband regions (Figure 3A), as has been suggested in previous reports (Dorsett et al., 2005; Gause et al., 2007; Markov et al., 2003). Several lines of evidence imply that these bands genuinely correspond to cohesin. First, coimmunostainings showed that myc-tagged Rad21^TEV^ is bound to the same chromosomal regions as endogenous Rad21 (Figure 3B). Second, cohesin's other three subunits (SMC1, SMC3, and SA/Scc3) colocalize with Rad21 on polytene-chromosome squashes (Figure S3A). Third, staining by myc-, Rad21-, and SMC1-specific antibodies is greatly reduced after TEV-induced cleavage of Rad21^TEV^ in flies in which this is the only form of Rad21 (Figure S3B). The fact that SMC proteins are also released implies that TEV cleavage of Rad21 releases the entire cohesin complex from chromosomes. Cohesin did not colocalize with known interband-specific proteins (Z4, BEAF32, Jil1, MSL2, CTCF), and its distribution differed significantly from numerous other proteins whose localization on polytene chromosomes has been well documented (PolII, Rpb3, HSF, trx, Pc, Su[Hw], CP190, Mod[mdg4]) (Figures S4A and S4B and data not shown). The cohesin holocomplex appears to be bound to distinct but as yet undefined regions of polytene chromosomes.

### Polytene Chromosomes Persist after Rad21 Cleavage

**Figure 4:**
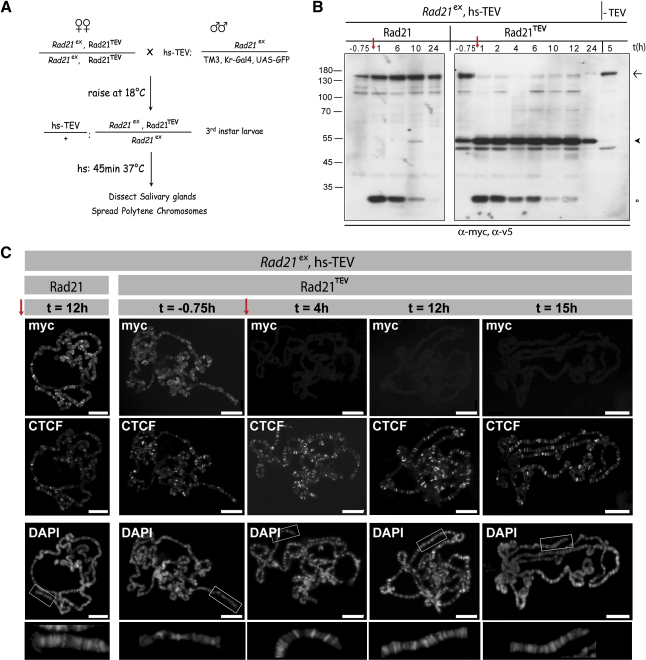
Cohesin Is Not Required for the Maintenance of Polytene-Chromosome Morphology (A) Outline of the TEV-cleavage experiment in salivary glands. (B) Western blot analysis of salivary gland extracts prepared either before (t = −0.75 hr) or at various time points after heat shock (red arrow) from GFP-negative larvae. The last lane shows a sample of salivary glands from Rad21^TEV^-expressing flies that do not contain hs-TEV. Blots were probed with antibodies against myc (detecting full-length transgenic Rad21 [arrow] and the C-terminal TEV-cleavage fragment [arrowhead]) and v5 (detecting TEV protease [open circle]). (C) Representative polytene-chromosome spreads of third-instar larvae that carry hs-TEV and express either transgenic Rad21 (left panel) or Rad21^TEV^ as their only source of Rad21 were prepared before (t = −0.75 hr) and at various time points after heat shock (red arrow). Polytene chromosomes were coimmunostained with antibodies against myc (recognizing Rad21) and CTCF. The morphology of the polytene chromosomes was visualized by DAPI staining (bottom row, higher magnification [2.5×]). All pictures were acquired by using the same acquisition settings. The scale bar is 20 μm.

To address whether cohesin holds polytene chromosomes together, we induced TEV by heat shock (from hs-TEV) in late third-instar larvae surviving on transgenic Rad21 with or without TEV-cleavage sites and containing morphologically normal polytene chromosomes (Figure 4A). After heat shock, TEV caused rapid cleavage of Rad21^TEV^ and its disappearance from polytene chromosomes for at least 15 hr, but it had no effect on Rad21 without TEV sites or on the staining pattern of CTCF, a boundary-binding factor (Moon et al., 2005) (Figures 4B and 4C). Surprisingly, the morphology of polytene chromosomes was unaltered by cohesin's removal (see DAPI stainings in Figure 4C), even when hypotonic or noncrosslinking conditions were used during spreading, which should favor their disassembly (data not shown). It is conceivable that the chromosomes retain their integrity by virtue of the small amount of full-length Rad21^TEV^ (Figure 4B) that persists after TEV cleavage (either due to resistance to TEV or due to Rad21 resynthesis). However, the simplest explanation for our results is that cohesin is not required for maintaining the integrity of polytene chromosomes.

Interestingly, cohesin is required for the normal development of salivary glands. In contrast to hs-TEV, which does not cause significant TEV expression at 18°C, leaky expression of TEV under the control of hs-Gal4/UAS at 18°C led to smaller salivary glands (∼1/2 the size) containing thinner polytene chromosomes in 100% of wandering late third-instar larvae that survived on Rad21^TEV^, as compared to controls (Figure S5). Importantly, this decrease in organ size was due to smaller, not fewer, cells per gland. Similar results were obtained by expressing TEV by using a salivary-gland-specific driver (F4-Gal4) (data not shown). These results suggest that cohesin has an essential function in nonproliferating, endocycling salivary gland cells.

### A Function for Cohesin in Neurons?

**Figure 5:**
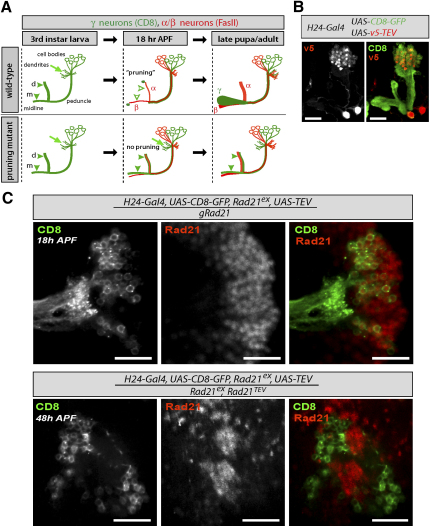
Cohesin Is Expressed in γ Neurons and Can Be Selectively Destroyed by TEV Cleavage (A) Schematic representation of axonal projections of γ (green) and α/β (red) neurons of wild-type and pruning-defective mutants at three characteristic time points during development. Only the right hemisphere is shown. α′/β′ neurons are omitted from the scheme. In third-instar larva, γ-neuron axons are bundled in the peduncle before they bifurcate to project into the dorsal (d) and medial lobes (m) (filled, green arrowheads). At 18 hr after puparium formation (APF), the dorsal and medial projections from wild-type γ neurons are selectively eliminated (“pruned,” open, green arrowheads). In a pruning mutant, γ-neuron axon projections and dendrites persist (filled, green arrowheads). α/β neurons project into the dorsal and medial lobes. In late pupae/adults, axons of wild-type γ neurons grow out again toward the midline. In a pruning mutant, larval axon projections of γ neurons persist in the dorsal and medial lobes. (B) H24-Gal4 was used to drive expression of v5-tagged nuclear TEV protease and mCD8 in γ neurons of the mushroom body. Third-instar larval brains were immunostained with antibodies against mCD8 (green) and the v5 epitope (red). Images show Z projections of single confocal sections of the right brain hemisphere. The scale bar is 20 μm. (C) H24-Gal4 was used to drive expression of TEV and mCD8 in γ neurons of the mushroom body from flies that expressed endogenous Rad21 (gRad21, top) or Rad21^TEV^ as their sole source of Rad21 (bottom). Brains were stained with antibodies against mCD8 (green) and Rad21 (red). Images show a single confocal section in the plane of γ-neuron cell bodies. Note that there is no overlap between the mCD8 and Rad21 stainings after TEV cleavage in γ neurons from Rad21^TEV^ flies. The scale bars are 20 μm.

The finding that cohesin is required for normal salivary gland development suggests that cohesin does indeed have nonmitotic functions. Because cohesin is essential for cell proliferation, its putative additional functions would be best studied in postmitotic cells that do not require chromosome segregation. This raises two key questions. First, is cohesin widely present in postmitotic cells in the fly, and, second, is it possible to use TEV-mediated Rad21 cleavage to inactivate the complex in such cells? The answer to both questions is yes. Immunostaining showed that Rad21 is concentrated within the nuclei of most neurons in larval brains (Figure 5C and data not shown). Moreover, expression of TEV in neurons from Rad21^TEV^-rescued flies during embryonic or larval development, by using the pan-neuronal drivers elav-Gal4 and nsyb-Gal4, causes developmental arrest and lethality (data not shown).

### Cohesin Rings Are Essential for Axonal and Dendritic Pruning

To investigate in more detail cohesin's function in neurons, we concentrated on postmitotic γ neurons in the mushroom body of the fly brain. We focused on these particular cells because a recent mosaic screen for *piggyBac* insertions that cause abnormal pruning of γ-neuron axons has implicated two other subunits of the cohesin complex, namely, SMC1 and SA/Scc3 (Schuldiner et al., 2008, this issue of *Developmental Cell*). γ neurons are a specific subtype of postmitotic neurons in the mushroom body of the fly brain. During larval stages, the axons of γ neurons project into the dorsal and medial lobes of the mushroom body. During metamorphosis, at the time when α/β neurons are born, larval γ-neuron projections are selectively eliminated in a process called “axonal pruning” (Figure 5A) (Lee et al., 1999; Watts et al., 2003).

We first addressed whether Rad21 is normally expressed in γ neurons. Immunostaining with Rad21-specific antibodies detected endogenous Rad21 within the nuclei of γ neurons and those of their neuronal neighbors (Figure 5C). TEV protease can be expressed in γ neurons via specific Gal4 driver lines (e.g., H24-Gal4) (Zars et al., 2000) and localizes to their nuclei (Figure 5B). Crucially, TEV expression in Rad21^TEV^-rescued flies largely eliminated Rad21^TEV^ from γ neurons, but not from interspersed neighboring neurons (Figure 5C). In contrast, it had no effect on endogenous Rad21, which is not susceptible to TEV-induced cleavage.

**Figure 6:**
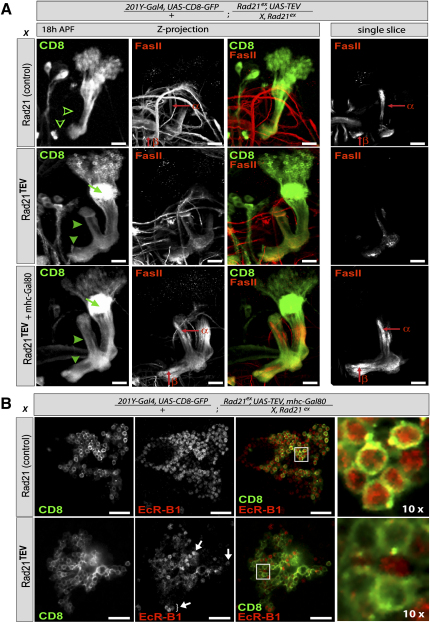
TEV Cleavage of Rad21 in γ Neurons Causes a Defect in Pruning (A and B) 201Y-Gal4 was used to drive expression of TEV and mCD8 in γ neurons of the mushroom body from flies that survived on transgenic Rad21 with or without TEV-cleavage sites. The scale bars are 20 μm. (A) Brains were dissected at 18 hr APF and were stained with antibodies against mCD8 (green) and FasII (red). Z projections of single confocal sections of the right brain hemisphere (left three panels). A single FasII-stained slice in the plane of α/β neurons (right panel). Absence/presence of γ-neuron projections (open/filled, green arrowheads), dendrites (green arrow), and α/β neurons (red arrows). In the bottom row, expression of Gal4 was suppressed in muscles by mhc-Gal80 in Rad21^TEV^ flies. (B) Brains of Rad21^TEV^ flies, in which Gal4 expression in muscles was suppressed by mhc-Gal80, were dissected at 18 hr APF and were stained with antibodies against mCD8 (green) and EcR-B1 (red). Images show single confocal sections in the plane of γ-neuron cell bodies. A higher-magnification view (10×) of the white-boxed area is shown on the right.

We next analyzed the consequences of cohesin cleavage. The driver line 201Y-Gal4 is expressed in mushroom-body γ neurons and has therefore been widely used in previous studies of the pruning process (Lee et al., 1999). In strains surviving on Rad21 without TEV sites and expressing 201Y-Gal4-driven TEV, the dendrites and axons of CD8-positive γ neurons and of FasII-positive α/β neurons were indistinguishable from wild-type. The axons of γ neurons initially projected into both dorsal and medial lobes (not shown) but were pruned by 18 hr after puparium formation (APF) (Figure 6A, pruned axons are indicated with open arrowheads). In Rad21^TEV^ larvae, γ neurons also projected their axons into dorsal and medial lobes (Figure S6A), but they failed to prune these projections during pupariation (Figure 6A, middle row). However, the absence of axons of later-born α/β neurons (with high levels of FasII) in the center of the dorsal and medial lobes at 18 hr APF (compare upper right panel to middle right panel in Figure 6A) suggests that pupae arrest early after pupariation, before α/β neurons are born. This raises the possibility that the pruning defect is in fact caused by arrest at a developmental stage that preceeds γ-neuron pruning.

Although specific for γ neurons within the central nervous system, the 201Y-Gal4 driver is also expressed in muscles (O.S. and L. Luo, unpublished data). The developmental arrest might therefore be caused by inactivation of cohesin in muscles. To test this, we expressed Gal80 under control of the muscle-specific myosin heavy-chain (mhc) promoter (C. Winter and L. Luo, personal communication) to prevent TEV expression and hence cohesin cleavage in muscles. Remarkably, this enabled pupae to develop well beyond the stage when pruning normally occurs. FasII-positive α/β neurons were now readily detected from 18 hr APF (Figure 6A, bottom panels). Because α/β neurons are descended from neuroblasts that proliferate after giving rise to γ neurons (Lee et al., 1999), the mere presence of α/β neurons implies that neuroblast proliferation is not blocked by the cleavage of Rad21 orchestrated by 201Y-Gal4. Importantly, the pruning defect in γ neurons caused by Rad21 cleavage was still observed (Figure 6A, bottom panels).

If the pruning defect of postmitotic γ neurons is caused by inactivation of cohesin in γ neurons themselves and is not an indirect consequence of its inactivation in some other cell type, then expression of TEV protease under control of a different γ-neuron-specific Gal4 driver should cause a similar phenotype. TEV expression via the H24-Gal4 driver confirmed that Rad21 cleavage in γ neurons causes axonal pruning defects (Figure S6B). Furthermore, comparison of γ-neuron projections between strains with and without cohesin in H24-Gal4-positive cells revealed that γ neurons also failed to prune their dendrites upon Rad21 cleavage (Figure S6B). Although we did not observe axon-targeting defects during larval and early pupal stages, the axonal projections of brains from late pupae (>4 days APF), which contain fully differentiated adult structures, were very often disorganized and mistargeted (Figure S6C). Our finding that a similar pruning defect accompanies Rad21 cleavage induced by two different Gal4 drivers, whose only common (known) feature is expression in γ neurons, implies that cohesin is needed for pruning of γ-neuron axons and dendrites.

How might cohesin regulate pruning? Previous work has implicated the ecdysone receptor EcR-B1 as a key regulator of γ-neuron pruning (Lee et al., 2000). Indeed, pruning defects caused by *SMC1* mutations are suppressed by overexpression of EcR-B1 (Schuldiner et al., 2008). The TEV-cleavage system should be ideal for testing whether cohesin is needed for EcR-B1 expression in all γ neurons. We found that Rad21 cleavage caused a major drop (at 18 hr APF) in the concentration of EcR-B1 within nuclei from most γ neurons, but not from nuclei of other interspersed neurons (Figure 6B). Only a minority of γ neurons still contained detectable levels of EcR-B1 upon Rad21 cleavage (indicated by white arrows). These data suggest that cohesin is required for cell-type-specific EcR-B1 expression.

### Cohesin Is Required in Cholinergic Neurons for Larval Locomotion

**Figure 7:**
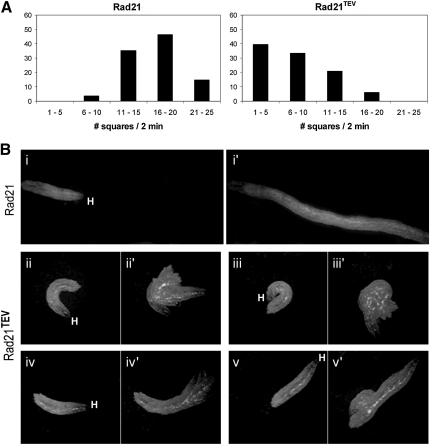
TEV Cleavage of Rad21 in Cholinergic Neurons Induces Severe Locomotion Defects in Third-Instar Larvae (A) Wandering third-instar larvae expressing TEV under the control of Cha-Gal4 and surviving on transgenic Rad21 with and without TEV sites were tested for motility (Rad21: *Cha-Gal4/+; Rad21^ex3^, Rad21-myc/Rad21^ex3^, UAS-TEV*; Rad21^TEV^: *Cha-Gal4/+; Rad21^ex15^, Rad21^TEV^/Rad21^ex3^, UAS-TEV*). Larval movements were tracked and superimposed to a grid. Locomotion was measured by the number of grid squares each larva traveled through. The number of larvae that traveled through the indicated number of squares (1–5, 6–10, etc.) is shown as a percentage of the total number of larvae tested (54 and 48 for strains containing Rad21 and Rad21^TEV^, respectively). (B) Representative images and temporal projections of movements from larvae that express TEV in cholinergic neurons and survive on either transgenic Rad21 (i and i′) or Rad21^TEV^ (ii–v′) (same genotypes as in [A]). (i)–(v) show the initial position of the larvae. H indicates the position of the head. (i′)–(v′) show the temporal projections of the images taken over a 20 s interval (images taken every 2 s). Note that controls move mostly straight, whereas larvae in which Rad21^TEV^ has been cleaved in cholinergic neurons show frequent episodes of turns, head movement, and backward motion.

One of the advantages of the TEV system is that it enables protein inactivation in all neurons of a given type and thereby has the potential to cause changes in animal behavior. To investigate this, we expressed TEV under control of Cha-Gal4, which expresses Gal4 specifically in cholinergic neurons (Salvaterra and Kitamoto, 2001). We noticed that this reduced the ability of Rad21^TEV^, but not Rad21, third-instar larvae to crawl up the sides of the vials. The larvae nevertheless pupariate, albeit within their food, and die as late pupae, with fully developed adult organs (data not shown). We used video imaging to compare locomotion of Rad21^TEV^ and transgenic Rad21 third-instar larvae after placing them in the center of a Petri dish containing nonnutritive agar. This revealed that larvae with TEV sites in Rad21 moved less far than those without (Figure 7A). More detailed analysis showed that larvae without TEV sites in Rad21 moved mostly in straight lines, whereas Rad21^TEV^ larvae curved repetitively (Figure 7Bii and 7Biii), frequently turned their heads (Figure 7Biv), and even moved backward (Figure 7Bv; see also Movies S5 and S6).

Three lines of evidence suggest that these dramatic changes are not caused by mitotic defects. First, chromosomes from brain cells expressing CD8-GFP driven by Cha-Gal4 were never positive for the mitosis-specific phosphohistone H3 marker (Figure S7A), implying that Cha-Gal4 does not drive expression in dividing cells. Second, brains from larvae surviving on Rad21^TEV^ and expressing TEV protease in cholinergic neurons do not have any detectable mitotic defects (<1%). Cohesion defects during mitosis would greatly delay passage through mitosis and therefore cause an increase in the percentage of phosphohistone H3-positive cells. No such effect was seen (Figure S7). Third, we were unable to detect any gross morphological defects in the pattern of cholinergic neurons marked by CD8-GFP driven by Cha-Gal4 or any reduction in their numbers, either in the central nervous system (Figure S7B) or in peripheral sensory organs (data not shown). We conclude that correct larval locomotion requires cohesin in cholinergic neurons.

## Discussion

### A Tool by Which to Study Protein Function in Metazoa

Although it was known that TEV protease can inactivate protein function in budding yeast (Uhlmann et al., 2000), it was unclear whether TEV could be used in a complex metazoan organism. Our work shows that TEV can be expressed in a wide variety of *Drosophila* tissues without causing overt toxicity. More important, we show that TEV expression induces quantitative cleavage of TEV-site-containing, but not wild-type, Rad21 protein, and that this is accompanied by penetrant phenotypes both in proliferating tissues and, more unexpectedly, in cells not engaged in mitosis, such as neurons and salivary gland cells.

The system we have developed has many attractive features that should make it a powerful and versatile tool for studying protein function in vivo. First, the method causes protein inactivation within a few hours and does not rely on a gradual depletion of the protein, as occurs in methods that interfere with the protein's synthetic capacity, such as recombinase-mediated gene deletion or RNA interference. Second, the system is reversible. By using Gal80ts, TEV protease can be turned both on and off. Third, it is possible to be certain that phenotypes are caused by cleavage of the target protein by comparing the effect of TEV expression in animals whose target protein either does or does not contain TEV sites. Fourth, by targeting the protease to particular locations inside or even (by using a secreted protease) outside cells, it should be possible to direct inactivation of the target protein to specific intra- or extracellular compartments. The restriction of protein inactivation to specific cellular compartments may be easier to devise by using TEV than degron systems relying on the much more complex process of ubiquitin-mediated proteolysis (Dohmen et al., 1994). Unlike the MARCM system, which uses FLP/FRT-induced mitotic recombination to generate homozygous mutant clones in proliferating tissues, TEV cleavage can be triggered in all cells of a given tissue and at any stage of development, features that will greatly facilitate phenotypic and biochemical analyses. Because many eukaryotic proteins contain multiple functional domains connected by unstructured polypeptide chains, protein inactivation through TEV cleavage should be applicable to a large variety of proteins. It could also be used to clip off protein domains and thereby alter protein activity.

### The Integrity of the Cohesin Ring Is Essential for Sister-Chromatid Cohesion in Mitosis

Our first priority upon developing a system to cleave Rad21 was to use it to investigate the role of cohesin during mitosis. In yeast, cohesin has a vital role in holding sister chromatids together until all chromosomes have bioriented during mitosis, whereupon cleavage of Scc1/Rad21 by separase triggers sister-chromatid disjunction (reviewed in Nasmyth and Haering, 2005). The consequences of depleting Scc1/Rad21 from tissue-culture cells by using RNA interference are, on the whole, consistent with the above-mentioned notion (Coelho et al., 2003; Vass et al., 2003). However, results from depletion experiments have not been able to directly explain the effects of inactivating cohesin within a single cell cycle.

We engineered a situation in which efficient cleavage of Rad21 occurred precisely as embryonic cells embarked on cycle 14, causing a devastating effect on mitosis. Chromosomes enter mitosis with paired sister kinetochores; however, instead of stably biorienting on a metaphase plate, they disjoin precociously, usually segregating to opposite poles. Importantly, these highly abnormal movements all take place prior to the APC/C-dependent activation of separase. These observations imply that cohesin is essential for the sister-chromatid cohesion necessary to resist mitotic-spindle forces in metazoan organisms as well as in yeast.

Our finding that most sister chromatids (in cells with cleaved Rad21) disjoin to opposite spindle poles, albeit precociously, suggests that their chromosomes possess sufficient cohesion to establish a transient form of biorientation, though possibly with low accuracy. We cannot at this stage determine whether this cohesion is mediated by cohesin complexes that have survived Rad21 TEV cleavage or by an independent cohesive mechanism such as residual sister DNA catenation. We can nevertheless conclude that the latter, if it exists, is incapable of resisting spindle forces and cannot therefore maintain sister-chromatid cohesion during a period in which the SAC has been activated and errors in chromosome biorientation are corrected. Thus, what really distinguishes cohesion mediated by cohesin from DNA catenation is its ability to be regulated by the SAC, and this may be the reason why eukaryotic cells appear to use cohesin for mitosis.

### The Cohesin Ring Has Key Functions in Nonmitotic Cells

Mutations in Scc2's human ortholog as well as in SMC1 and SMC3 cause the developmental defects associated with CdLS (reviewed in Dorsett, 2007). It is unclear whether these defects are caused by mitotic errors during development or by defects in nonmitotic cohesin functions. The first clue that cohesin might indeed play key roles during development other than holding sister chromatids together was the finding that mutations in *D*. *melanogaster* *Nipped-B*, the ortholog of *Scc2*, alters the expression of genes whose regulatory sequences have been mutated (Rollins et al., 1999).

If cohesin has nonmitotic functions during development, then these could occur in proliferating and nonproliferating (postmitotic) cells. To analyze cycling cells, it would be necessary to restrict analysis either to a short, specific cell-cycle stage (e.g., the G1 period) or to develop a means of differentially inactivating cohesin complexes engaged in nonmitotic functions, leaving intact those engaged in chromosome segregation. Analysis of postmitotic cells is easier. It is merely necessary to devise a protocol for inactivating cohesin only after cell proliferation has ceased.

Cleavage of Rad21 induced by postmitotic pan-neuronal drivers caused lethality, suggesting that cohesin has key functions in neurons. To investigate these in greater detail, we analyzed the effects of Rad21 cleavage in specific neuronal subtypes. The finding that the proliferative defects caused by a *SMC1* mutation in clones of mushroom-body neuroblasts are accompanied by defective pruning of axons (Schuldiner et al., 2008) led us to investigate the effects of Rad21 cleavage in γ neurons. Our results show that Rad21 cleavage abolished the developmentally controlled pruning of both axons and dendrites in γ neurons. These defects cannot have been caused by failures in cell division because cleavage had no effect on the birth of γ neurons or on their initial axonal projections.

Previous work on *mau-2* (the *C. elegans* Scc4 ortholog) has already provided a link between cohesin and axon development (Benard et al., 2004). Whereas Mau-2 was reported to act as a guidance factor required for correct axon and cell migration, investigation of γ neurons in *Drosophila* suggests that cohesin mediates the elimination of axon projections and dendrites. However, our results do not rule out a function for cohesin in regulating axon guidance because Rad21 cleavage might not be complete when γ-neuron axons start growing out in the first place. Indeed, we observed axon-projection defects in developmentally arrested late pupae.

It has not thus far been possible to show that γ-neuron pruning defects cause changes in animal behavior. Cleavage of cohesin in the entire population of cholinergic neurons, in contrast, has a dramatic effect, causing larvae to turn frequently, move their heads back and forth, and even crawl backward. Importantly, the neurons clearly survive without functional cohesin and must be at least partially active, because larvae are not paralyzed by cohesin cleavage, a phenotype seen when cholinergic transmission is switched off (Kitamoto, 2001). The locomotion defects are not dissimilar to those caused by mutations in *scribbler* (*sbb*) (Yang et al., 2000). *sbb*, also known as *brakeless* (*bks*) and *master of thickveins* (*mtv*), codes for a ubiquitously expressed corepressor of transcription (Haecker et al., 2007 and references therein). Expression of a *sbb* transcript exclusively in cholinergic neurons is sufficient to rescue locomotion defects of *sbb* mutants (Suster et al., 2004). It therefore appears that the lack of *sbb* and cohesin in cholinergic neurons causes similar locomotion defects. Future work will have to show whether there is a link between *sbb* and cohesin. Our finding that cohesin has roles in neurons that are essential for normal behavior is consistent with the notion that the mental retardation invariably found in patients with CdLS is also due to defective neuronal function, as opposed to defective cell proliferation during development.

We have shown that suppression of 201Y-Gal4-induced TEV expression, specifically in muscles, bypasses the early pupal arrest in Rad21^TEV^-rescued flies and indicates that cohesin is essential in muscles as well as in neurons. In addition, although cohesin does not seem to be required for the maintenance of polytene-chromosome morphology, it is essential for normal progression through the endocycle in salivary glands. It is therefore conceivable that cohesin has key functions in most postmitotic cell types. What might these functions be? Cohesin is known to be required for efficient double-strand break repair as well as sister-chromatid cohesion (reviewed in Nasmyth and Haering, 2005), and it promotes repair by facilitating homologous recombination between sister chromatids. Its action in postmitotic neurons, however, must be on unreplicated chromatids. We suggest therefore that cohesin's function in neurons and other postmitotic G0 cells is more likely to be in regulating gene expression. The finding that cohesin cleavage reduces the accumulation of EcR-B1 within γ neurons is consistent with this notion. Interestingly, recent data have shown that cohesin binds to the EcR gene in several fly cell lines (Misulovin et al., 2007). Future experiments should address whether cohesin acts as a general regulator of gene expression.

In summary, we provide definitive evidence that the cohesin ring has essential functions in cells with unreplicated chromosomes. It will be important in the future to establish whether cohesin functions by trapping chromatin fibers, as it appears to do in cells that have replicated their genomes.

## Experimental Procedures

### Fly Strains

TEV-cleavage experiments were performed in a *Rad21* null background. Four independent *Rad21^ex^* alleles were generated by imprecise excision of the P element *GE50159* (see the Supplemental Data for details). For the generation of transgenic flies expressing TEV-cleavable versions of Rad21 under control of the tubulin-promotor (Rad21^TEV^), three TEV-recognition sites were introduced into a previously generated *pCaSpeR-Rad21-myc_10_* vector. To generate a nuclear v5-tagged TEV protease expression construct, three NLS- and one v5-epitope tag were added to the coding region of TEV. For cloning details, see the Supplemental Data. Transgenic lines were produced by standard P-element-mediated germline transformation.

The fly stock *Rad21^ex15^, Rad21(550-3TEV)-myc* was used as a source of TEV-cleavable Rad21 (Rad21^TEV^). The only exceptions are the western blot experiment in Figure 1D and the characterization of the zygotic Rad21 mutant phenotype (Figure 2, Figure S2, Movies S1–S4), for which *Rad21^ex8^, Rad21(271-3TEV)-myc* and *2x Rad21(271-3TEV)-myc; Rad21^ex3^*, respectively, were used as sources of Rad21^TEV^.The fly stock *Rad21^ex3^, Rad21-myc* served as a control (transgenic Rad21 without TEV sites).

Further details on stocks can be found in the Supplemental Data. A complete stocklist with all genotypes and abbreviations used in the text can be found in Table S1.

### Immunofluorescence of Embryos after TEV Cleavage of Rad21

For analysis of mitosis after TEV-induced cleavage of Rad21^TEV^ in fixed samples, 3–6 hr embryos were collected from a cross between *α4-tub-Gal4/2x Rad21(271-3TEV); Rad21^ex3^* females and *UAS-TEV, hs-Gal4, Rad21^ex3^/TM3, Kr-Gal4, UAS-GFP* males. Immunofluorescence labeling of embryos was performed according to standard procedures (Knoblich and Lehner, 1993) after a preincubation in 0.7 μM taxol before fixation. Pictures were acquired with a Zeiss Axioplan 2 imaging system by using the Zeiss AxioVision software. The following initial experiment allowed us to distinguish between +TEV and −TEV embryos: *α4-tub-Gal4/2x Rad21(271-3TEV); Rad21^ex3^* females were crossed to either *UAS-TEV, hs-Gal4, Rad21^ex3^/TM3, Kr > GFP* (+TEV) or *hs-Gal4, Rad21^ex3^/TM3, Kr > GFP* (−TEV) males. Embryos were fixed during mitosis 14 and were stained with anti-tubulin and a DNA stain. 50% of the embryos from the first cross displayed a drastic mitotic delay, whereas the other 50% were phenotypically wild-type. In contrast, all embryos from the second cross were phenotypically wild-type. These observations indicate that +TEV embryos can be identified readily based on their severe mitotic abnormalities.

### In Vivo Imaging of Embryos after TEV Cleavage of Rad21

For in vivo imaging of mitosis after TEV-induced cleavage of Rad21, Rad21^TEV^-rescued flies that contained fluorescent markers for DNA (His2Av-mRFP1) and kinetochores (EGFP-Cid) as well as the maternal Gal4 driver α4-tub-Gal4 on their second chromosome were generated. *α4-tub-Gal4, His2Av-mRFP1, EGFP-Cid/2x Rad21(271-3TEV)-myc; Rad21^ex3^* females were crossed with *UAS-TEV, hs-Gal4, Rad21^ex3^/TM3, Kr-Gal4, UAS-GFP* males. Embryos of this cross either displayed the characteristic severe mitotic abnormalities and were thus considered to be TEV expressing (+TEV) or they were entirely normal and thus considered to lack the UAS-TEV transgene (−TEV). In vivo imaging was performed essentially as described (Schuh et al., 2007). Time-lapse confocal laser scanning microscopy was performed with an inverted Leica TCS SP1 system equipped with a 40×/1.25 oil immersion objective at 22°C–24°C. One stack of five frames was acquired every 15 s. The Leica confocal software was used for maximum projection, Gaussian filtering, and contrast adjustment.

### Immunoblotting

Pupae or dissected salivary glands were homogenized in SDS-sample loading buffer and boiled for 5 min. Western blot was performed according to standard procedures.

### Immunostaining of Polytene-Chromosome Squashes

Polytene-chromosome spreads were prepared according to standard procedures as outlined in the Supplemental Data. Fluorescent images were acquired with an AXIO Imager.Z1 microscope (Zeiss) and a CoolSNAP HQ CCD camera (Photometrics) by using MetaMorph software (Universal Imaging).

### Immunostaining of Brains

Immunostaining of whole-mount brains was performed as described previously (Lee and Luo, 1999). Confocal pictures were obtained by using a Zeiss LSM 510 Axiovert 200M. Maximal projections of Z stacks were generated by using Zeiss software.

### Antibodies

The following primary antibodies were used (WB, western blot; IF, immunofluorescence): guinea-pig α-Rad21 (WB, 1:3000, IF, 1:600) (Heidmann et al., 2004), mouse α-myc 9E10 (WB, 1:200; Sigma-Aldrich), mouse α-myc 4A6 (IF, 1:500; Upstate), mouse α-v5 (WB, 1:5000, IF, 1:500; Invitrogen), mouse α-Cyclin B (F2) (1:3) (Knoblich and Lehner, 1993), mouse α-tubulin (DM1A) (1:8000; Sigma-Aldrich), rabbit α-BubR1 (1:2000) (Logarinho et al., 2004), rabbit α-CTCF (1:200) (Moon et al., 2005), rat α-mCD8 α subunit (1:100; Abcam), mouse α-FasII (1D4) (1:50; Developmental Studies Hybridoma Bank [DSHB]), mouse α-EcR-B1 (AD4.4) (1: 25; DSHB), rabbit α-phosphohistone H3 (1:500; Upstate), and rabbit α-actin (1:1000; Abcam). For WB, HRP-linked secondary antibodies (Amersham) were detected by Enhanced Chemi-Luminescence (ECL) (Amersham). For IF, Alexa-conjugated secondary antibodies (Molecular Probes) were used as 1:500 dilutions.

### Larval Behavior

Larval locomotion was tested essentially as previously described (Yang et al., 2000), with minor modifications. Late third-instar larvae of control strains were selected based on their characteristic wandering stage. Since Rad21-depleted larvae do not crawl up the walls of food vials, Rad21^TEV^ larvae were considered as “wandering” based on their size and the fact that they stopped foraging within the food. “Wandering” third-instar larvae were placed in the center of 90 mm diameter Petri dishes coated with nonnutritive 2% agar. After 1 min of adaptation, the movement was recorded over a period of 2 min by taking images every 5 s with a Canon Power Shot S70 digital camera. Movies were assembled, and larval movement was manually tracked by using ImageJ 1.38× software. Total locomotion was measured by superimposing trails onto a 6 mm grid and counting the number of squares through which larvae moved. For detailed analysis of locomotion behavior, higher magnification movies were taken on a dissection scope coupled to a Canon Power Shot S70 digital camera. For temporal projection of larval movement, single images were extracted from the recorded movies with a time lapse of 2 s. Projections of 10 images (corresponding to 20 s periods) were obtained by using ImageJ software.

## Supplemental Data

Document S1. Supplemental Experimental Procedures, Two Tables, Seven Figures, and Supplemental References

Movie S1 Progression through mitosis 14 of *Drosophila* embryogenesis is illustrated as observed by time-lapse in vivo imaging of a control embryo expressing His2Av-mRFP1 and EGFP-Cid, but not UAS-TEV. A maximum projection of five confocal sections with 600 nm step size acquired at 15 second intervals using a 100× (NA 1.4) objective is presented. Several epidermal cells enter and progress through mitosis 14. Occasional lateral jumps in the movie reflect periodic stage movements for compensation of the embryonic germband extension movements.

Movie S2 Progression into mitosis 14 after Rad21^TEV^ cleavage by zygotic UAS-TEV expression. Time-lapse in vivo imaging of an embryo expressing His2Av-mRFP1 and EGFP-Cid was performed. Technical details were as described for Supplemental Movie 1. Two cells enter mitosis soon after the start of the movie followed by premature separation of sister chromatids.

Movie S3 Chromatid behavior during the mitotic arrest in cycle 14 after cleavage of Rad21^TEV^ by zygotic UAS-TEV expression. Time-lapse in vivo imaging of an embryo expressing His2Av-mRFP1 and EGFP-Cid was performed. Five confocal sections with 600 nm step size were acquired at 15 second intervals using a 100× (NA 1.4) objective. Single sections are shown in the acquired sequence. One of the separated chromatids loses its orientation to the left pole and is reoriented to the opposite pole on the right.

Movie S4 Adaptation and exit from the mitotic arrest resulting from cleavage of Rad21^TEV^ by zygotic UAS-TEV expression. Time-lapse in vivo imaging of an embryo expressing His2Av-mRFP1 and EGFP-Cid was performed. A maximum projection of five confocal sections with 600 nm step size acquired at 15 second intervals using a 40× (NA 1.25) objective is presented. Epidermal cells in the upper half of the movie have already entered mitosis 14 and are in a mitotic arrest at the start of the movie. Several cells eventually adapt and exit from mitosis.

Movie S5 Movie showing the locomotion of a third-instar control larva (genotype: *Cha-Gal4/+; Rad21^ex3^, Rad21/Rad21^ex3^, UAS-TEV*) on nonnutritive agar.

Movie S6 Movie showing the locomotion of a third-instar larva after TEV cleavage of Rad21^TEV^ in cholinergic neurons (genotype: *Cha-Gal4/+; Rad21^ex15^, Rad21^TEV^/Rad21^ex3^, UAS-TEV*).
