# Supplementary material for: Cell-Type-Specific TEV Protease Cleavage Reveals Cohesin Functions in Drosophila Neurons
Source: Dev Cell. 2008 Feb 12;14(2):239–51. doi: 10.1016/j.devcel.2007.12.009 (PMC2258333; doi:10.1016/j.devcel.2007.12.009)
Supplement: Document S1. Supplemental Experimental Procedures, Two Tables, Seven Figures, and Supplemental References [file mmc1.pdf]

Developmental Cell, volume 14

Supplemental Data

Cell-Type-Specific TEV Protease Cleavage Reveals Cohesin Functions in *Drosophila* Neurons

Andrea Pauli, Friederike Althoff, Raquel A. Oliveira, Stefan Heidmann, Oren Schuldiner, Christian F. Lehner, Barry J. Dickson, and Kim Nasmyth

Supplemental Experimental Procedures

Fly stocks

The line *P{w*$^{+}$*, EP}GE50159* was obtained from GenExel, Korea. Deficiencies in heterochromatin 3 and the *α4-tub-Gal4* driver line are available from the Bloomington stock center. Transgenic lines with *His2Av-mRFP1, EGFP-Cid* (Schuh et al., 2007), *gSMC3-HA* (Heidmann et al., 2004), *201Y-Gal4* (Yang et al., 1995), *H24-Gal4* (Zars et al., 2000) and *Cha-Gal4* (Salvaterra and Kitamoto, 2001) have been described previously. A *mhc-Gal80* fly stock (C. Winter and L. Luo, unpublished) was kindly provided by Liqun Luo. A complete stocklist with all genotypes and abbreviations used in this paper can be found in Supplemental Table S1.

Generation of *Rad21* alleles

The insertion site of the homozygous viable *P{w*$^{+}$*, EP}GE50159* line 4 kb upstream of the transcription start site of *Rad21* was confirmed by inverse PCR according to a standard protocol. Imprecise excisions were generated by crossing the GE50159 line to flies expressing a stable source of the P-element transposase. Out of 500 excision events, 23 homozygous lethal lines were isolated. 4 independently generated deletions affecting *Rad21* were subsequently identified by PCR (*Rad21*^{ex3}, *Rad21*^{ex8}, *Rad21*^{ex15}, *Rad21*^{ex16}) and confirmed by sequencing DNA fragments spanning the breakpoints.

Generation of transgenic flies expressing TEV protease

To generate a NLS- and v5-epitope-tagged TEV protease expression construct (nuclear TEV protease), one N-terminal consensus sequence of the SV40 nuclear localization signal (NLS) followed by one v5-epitope tag were introduced by PCR at the 5'end of the coding sequence of TEV-NLS_2, using the yeast vector YIplac204 (Uhlmann et al., 2000) as template. Primer sequences are listed below. The PCR product *NLS-v5-TEV-*NLS_2 was cloned as EcoRI/NotI fragment into the pUAST (Brand and Perrimon, 1993) or pCaSpeR-hs (Thummel, 1992) transformation vectors to obtain *UAS-NLS-v5-TEV-*NLS_2 or *hs-NLS-v5-TEV-*NLS_2, respectively.

Transgenic lines were produced by standard P-element-mediated germline transformation (Rubin and Spradling, 1983) and either recombined to the $Rad21^{ex3}$ allele ($Rad21^{ex3}$*, UAS-TEV/TM3, Sb, Kr-Gal4, UAS-GFP*) or crossed into the $Rad21^{ex3}$ background (*hs-TEV;* $Rad21^{ex3}$*/TM6B, Tb*).

Generation of flies surviving on transgenic $\mathbf{Rad21^{TEV}}$

C-terminally 10xmyc-tagged Rad21 was created based on the EST clone LD14219 obtained from the Berkeley Drosophila Genome Project (BDGP). The coding sequence of the 10xmyc-tag was amplified by PCR from the plasmid *gthr-myc* (Leismann et al., 2000) and cloned as Bst-BI fragment into the unique Bst-BI site in LD14219, located 12 nucleotides upstream of the Rad21 translational stop codon. The sequence encompassing *Rad21-*myc_{10} was excised as a 2790 bp Eco-RI//Kpn-I fragment and cloned into pUAST (Brand and Perrimon, 1993) to obtain *pUAS-Rad21-*myc_{10}.

To generate TEV-cleavable versions of Rad21 (Rad21^{TEV}), SpeI-restriction sites were introduced into the coding region of *Rad21* after amino acids 175, 197, 271 or 550 by site-directed PCR-mutagenesis, using *pUAS-Rad21-myc*$_{10}$ as template (primer sequences see below). An AvrII/NheI restriction fragment encoding 3 tandem arrays of the TEV-recognition sequence ENLYFQG (kindly provided by Stephan Gruber, for details on the sequence see below) was inserted into the newly generated SpeI-site. The *Rad21(3TEV)-myc*$_{10}$ fragment (EcoRI/KpnI-blunt) was introduced into the multiple cloning site (MCS) of a modified EcoRI/blunt cut pBS vector containing a MCS and 3'UTR flanked by FRT-sites (<). Next, the sequence comprising <*Rad21(3TEV)-myc*$_{10}$*-3'UTR*< was excised with KpnI and inserted into a KpnI-cut modified pCaSpeR transformation vector (derived from 10xUAS, G. Dietzl), in which the 10xUAS-sequence had been replaced with the sequence of the tubulin-promotor (derived from plasmid M>P^2, Casali and Struhl, 2004) (final vectors: *tubpr*<*Rad21(3TEV)-myc*$_{10}$*-3'UTR*<*SV40).*

To generate vectors expressing myc-tagged Rad21 without TEV-cleavage sites, the open reading frame of *Rad21-myc*$_{10}$ was PCR-amplified from *pUAS-Rad21-myc*$_{10}$ and introduced into the same modified vector (final vector: *tubpr-Rad21-myc*$_{10}$*-SV40).*

Transgenic lines were produced by standard P-element-mediated germline transformation (Rubin and Spradling, 1983). Transgenes were tested for their ability to rescue the lethality of *Rad21* null mutations. Transgenes with TEV-cleavage sites at positions 271 or 550 and a transgene without TEV-sites were functional.

Immunoblotting

For the preparation of embryonic extracts, dechorionated embryos were homogenized in SDS-sample loading buffer 3-6 hours after egg deposition. Proteins were resolved by SDS-polyacrylamide gel electrophoresis and transferred to a PVDF membrane. The blot was probed with mouse anti-myc 9E10 (1:15, Sigma-Aldrich) and mouse anti-α-tubulin (DM1A) (1:20000, Sigma-Aldrich) using ECL detection (Amersham). Protein extracts from pupae, 3rd instar larval salivary glands and 3rd instar larvae without salivary glands were prepared after dissection in PBS essentially as described in Experimental Procedures.

Immunostaining of polytene chromosome squashes

Salivary glands from wandering 3rd instar larvae were dissected in PBS, permeabilized in PBX* (PBS, 0.1% Triton X-100, 3.7% formaldehyde) for 30 seconds, fixed in 45% acetic acid/3.7% formaldehyde for 5-7 minutes and squashed according to standard procedures. Slides were blocked for 1 hour at room temperature in PBST + 5% BSA (PBS, 0.01% Tween20, 5% BSA) and incubated with primary antibodies (diluted in blocking solution) overnight at 4°C. After washing in PBST (3x10 minutes), slides were incubated with Alexa-conjugated secondary antibodies at room temperature for 1.5 hours, washed as before and mounted using VECTASHIELD mounting medium containing DAPI (Vector Laboratories).

The following (additional) primary antibodies were used: guinea-pig anti-SA (1:100, Dorsett et al., 2005), rabbit anti-SMC1 (1:100, Dorsett et al., 2005), rat anti-SMC1 (1:500, Malmanche et al., 2007), mouse anti-HA 16B12 (1:250, Covance), mouse anti-polymerase II 8WG16 (1:20, Covance), rabbit anti-trx (1:50, Chinwalla et al., 1995), rabbit anti-Pc (1:200, Zink and Paro, 1989), rabbit anti-HSF (1:80, Andrulis et al., 2000), rabbit anti-BEAF32 (SCBP) (1:50, Zhao et al., 1995) and mouse anti-Z4

(1:1, Saumweber et al., 1980).

Primer and DNA sequences

Restriction enzyme sites are shown in lower case, the start codon **ATG** in bold, the *v5*-epitope sequence in *italics*, the NLS sequence underlined.

NLS-v5-TEV-NLS$_2$

AP2f 5’-AAAgaattcAAA**ATG***CCTAAGAAAAAGAGGAAGGTT*GCATCCGGTAAGCCTATCCCTAACCCTCTCCTCGGTCTCGATTCTACGGGAGAAAGCTTGTTTAAGGGACCACG-3’

AP4r 5’-AAAAACCCTCGAGCCgcggccgcAG-3’

pUAS-Rad21-myc$_{10}$

SH28 5’-CGATTATTCGAAAACCCAAAAATTGTTCGCCATGGTATGG-3’

SH29 5’-CGATTA TTCGAA TGCCCCATGTTCGCCCAAG-3’

tubpr-Rad21-myc$_{10}$* and *tubpr<Rad21(3TEV)-myc$_{10}$-3’UTR<

AP28-EcoRIRad21f 5’-AAAgaattcAAA**ATG**TTCTATGAGCACATTATTTTG-3’

AP29-Rad21NotIr 5’-AAAgcggccgcATTAAAACAGATTTACATTCAAC-3’

AP20-TEV175SpeIf 5’-CCTCTATTTTCAAGGCactagtATACCTTCAAATATTAATGATAAA-3’

AP21-175NheISpeIr 5’-actagtGCCTTGAAAATAGAGGTTCTCGCTAGCTTCAAAGCCTATATCACCAAA-3’

AP22-197SpeIf 5’-TTTTGGAAAATATTGAGactagtTCTCTGGATCCACATTCATTGG-3’

AP23-197SpeIr 5’-actagtCTCAATATTTTCCAAAACGTC-3’

AP24-271SpeIf 5’-CATAATGTCCCTTCGCCTactagtGCAACCTCGCTCGTTAATTCGATTG-3’

AP25-271SpeIr 5’-actagtAGGCGAAGGGACATTATGAATATT-3’

AP26-550SpeIf 5’-TCAAGGAGACTCAACGactagtCCAGCTGGGTTGGATCATGGTC-3’

AP27-550SpeIr 5’-actagtCGTTGAGTCTCCTTGATTAAAA-3’

3 TEV recognition sites (3TEV)

c/ctagGGCTAGAGAGAATTTGTATTTTCAGGGTGCTTCTGAAAACCTTTACT

TCCAAGGAGAGCTCGAAAATCTTTATTTCCAGGGAg/ctagc

protein sequence: "-RAR**ENLYFQG**AS**ENLYFQG**EL**ENLYFQG**AS-"

Supplemental References

Andrulis, E.D., Guzman, E., Doring, P., Werner, J., and Lis, J.T. (2000). High-resolution localization of Drosophila Spt5 and Spt6 at heat shock genes in vivo: roles in promoter proximal pausing and transcription elongation. Genes Dev *14*, 2635-2649.

Brand, A.H., and Perrimon, N. (1993). Targeted gene expression as a means of altering cell fates and generating dominant phenotypes. Development (Cambridge, England) *118*, 401-415.

Casali, A., and Struhl, G. (2004). Reading the Hedgehog morphogen gradient by measuring the ratio of bound to unbound Patched protein. Nature *431*, 76-80.

Chinwalla, V., Jane, E.P., and Harte, P.J. (1995). The Drosophila trithorax protein binds to specific chromosomal sites and is co-localized with Polycomb at many sites. Embo J *14*, 2056-2065.

Dorsett, D., Eissenberg, J.C., Misulovin, Z., Martens, A., Redding, B., and McKim, K. (2005). Effects of sister chromatid cohesion proteins on cut gene expression during wing development in Drosophila. Development (Cambridge, England) *132*, 4743-4753.

Heidmann, D., Horn, S., Heidmann, S., Schleiffer, A., Nasmyth, K., and Lehner, C.F. (2004). The Drosophila meiotic kleisin C(2)M functions before the meiotic divisions. Chromosoma *113*, 177-187.

Leismann, O., Herzig, A., Heidmann, S., and Lehner, C.F. (2000). Degradation of Drosophila PIM regulates sister chromatid separation during mitosis. Genes Dev *14*, 2192-2205.

Malmanche, N., Owen, S., Gegick, S., Steffensen, S., Tomkiel, J.E., and Sunkel, C.E. (2007). Drosophila BubR1 is essential for meiotic sister-chromatid cohesion and maintenance of synaptonemal complex. Curr Biol *17*, 1489-1497.

Rubin, G.M., and Spradling, A.C. (1983). Vectors for P element-mediated gene transfer in Drosophila. Nucleic acids research *11*, 6341-6351.

Salvaterra, P.M., and Kitamoto, T. (2001). Drosophila cholinergic neurons and processes visualized with Gal4/UAS-GFP. Brain research *1*, 73-82.

Saumweber, H., Symmons, P., Kabisch, R., Will, H., and Bonhoeffer, F. (1980). Monoclonal antibodies against chromosomal proteins of Drosophila melanogaster: establishment of antibody producing cell lines and partial characterization of corresponding antigens. Chromosoma *80*, 253-275.

Schuh, M., Lehner, C.F., and Heidmann, S. (2007). Incorporation of Drosophila CID/CENP-A and CENP-C into centromeres during early embryonic anaphase. Curr Biol *17*, 237-243.

Thummel, C.S. (1992). Mechanisms of transcriptional timing in Drosophila. Science *255*, 39-40.

Uhlmann, F., Wernic, D., Poupart, M.A., Koonin, E., and Nasmyth, K. (2000). Cleavage of cohesin by the CD clan protease separin triggers anaphase in yeast. Cell *103*, 375-386.

Weiss, A., Herzig, A., Jacobs, H., and Lehner, C.F. (1998). Continuous Cyclin E expression inhibits progression through endoreduplication cycles in Drosophila. Curr Biol *8*, 239-242.

Yang, M.Y., Armstrong, J.D., Vilinsky, I., Strausfeld, N.J., and Kaiser, K. (1995). Subdivision of the Drosophila mushroom bodies by enhancer-trap expression patterns. Neuron *15*, 45-54.

Zars, T., Fischer, M., Schulz, R., and Heisenberg, M. (2000). Localization of a short-term memory in Drosophila. Science *288*, 672-675.

Zhao, K., Hart, C.M., and Laemmli, U.K. (1995). Visualization of chromosomal domains with boundary element-associated factor BEAF-32. Cell *81*, 879-889.

Zink, B., and Paro, R. (1989). In vivo binding pattern of a trans-regulator of homoeotic genes in Drosophila melanogaster. Nature *337*, 468-471.

Supplemental Tables

Table S1: Strains used in this study

Genotype	Abbreviation	Source
w^{1118}	*w*	Bloomington stock centre
w; lethal/CyO; TM2, {Δ2-3}/Sb, {Δ2-3}	P-element transposase	kindly provided by Frank Schnorrer
w; P{w+, EP}GE50159 (III)	*GE50159*	GenExel, Korea
**Gal4 driver**		
w; act-Gal4/CyO	*act-Gal4*	Barry Dickson lab stocks
w; hs-Gal4 (III)	*hs-Gal4*	Barry Dickson lab stocks
w; P{w+, mata4-tub-Gal4-VP16}	*a4-tub-Gal4*	Bloomington stock centre
w; P{w+, GawB}F4 (II)*	*F4-Gal4*	Weiss et al., 1998
w; elav-Gal4 (3AF) (III)	*elav-Gal4*	Barry Dickson lab stocks
w; nsyb-Gal4/CyO	*nsyb-Gal4*	kindly provided by Julie Simpson
y, w; 201Y-Gal4, UAS-mCD8-GFP/CyO; MKRS, Sb/TM6B, Tb	*201Y-Gal4, UAS-mCD8-GFP*	Liqun Luo lab stocks
w; Pin/CyO; H24-Gal4, UAS-mCD8-GFP	*H24-Gal4, UAS-mCD8-GFP*	Liqun Luo lab stocks
w; Cha-Gal4 (II)	*Cha-Gal4*	Salvaterra and Kitamoto, 2001
**Transgenes and Mutants**		
w; P{w+, His2Av-mRFP1} (II.2)*	*His2Av-mRFP1*	Schuh et al., 2007
w; P{w+, EGFP-Cid} (II.1)*	*EGFP-Cid*	Schuh et al., 2007
w; P{w+, gSMC3-HA12} (III.2)*	*gSMC3-HA*	Heidmann et al., 2004
y, w; P{w+, mhc-Gal80} (III)	*mhc-Gal80*	C. Winter and L. Luo, unpublished
Df(3L)2-66, kni[ri-1] p[p]/TM3, Sb, Ser	*Df(3L)2-66*	Bloomington stock centre
**Rad21-excisions**		
w; $Rad21^{ex3}$*/TM3, Sb, Kr-Gal4, UAS-GFP*	$Rad21^{ex3}$	present study
w; $Rad21^{ex8}$*/TM3, Sb, Kr-Gal4, UAS-GFP*	$Rad21^{ex8}$	present study
y, w; $Rad21^{ex15}$*/TM3, Sb, Kr-Gal4, UAS-GFP*	$Rad21^{ex15}$	present study
w; $Rad21^{ex16}$*/TM3, Sb, Kr-Gal4, UAS-GFP*	$Rad21^{ex16}$	present study

**TEV protease transgenes**		
w; P{w+, UAS-NLS-v5-TEV-NLS2} (III)	*UAS-TEV*	present study
w; P{w+, hs-NLS-v5-TEV-NLS2} (II)	*hs-TEV*	present study
**Rad21-excision + TEV-protease**		
w; Rad21^{ex3}*, P{w+, UAS-NLS-v5-TEV-NLS2}/TM3, Sb, Kr-Gal4, UAS-GFP*	*Rad21*^{ex3}*, UAS-TEV/TM3, Sb, Kr>GFP*	
w; Rad21^{ex3}*, P{w+, UAS-NLS-v5-TEV-NLS2}, hs-Gal4/TM3, Sb, Kr-Gal4, UAS-GFP*	*Rad21*^{ex3}*, UAS-TEV, hs-Gal4/TM3, Sb, Kr>GFP*	present study
w; P{w+, hs-NLS-v5-TEV-NLS2 }; Rad21^{ex3}*/TM6B, Tb, ubiquitin-GFP*	*hs-TEV ; Rad21*^{ex3}*/TM6B, Tb*	present study
**transgenic Rad21 (+/- TEV-sites)**		
w; P{w^{+}*, tubpr<Rad21(550-3TEV)-myc*$_{10}$*<SV40}* (III)	*Rad21(550-3TEV)-myc (Rad21*^{TEV}*)*	present study
w; P{w^{+}*, tubpr<Rad21(271-3TEV)-myc*$_{10}$*<SV40}* (III)	*Rad21(271-3TEV)-myc (Rad21*^{TEV}*)*	present study
w; P{w^{+}*, tubpr-Rad21-myc*$_{10}$*-SV40}* (III)	*Rad21-myc (Rad21)*	present study
w; P{w^{+}*, tubpr<Rad21(271-3TEV)-myc*$_{10}$*<SV40}* (II.3)	*Rad21(271-3TEV)-myc (Rad21*^{TEV}*)*	present study
w; P{w^{+}*, tubpr<Rad21(271-3TEV)-myc*$_{10}$*<SV40}* (II.7)	*Rad21(271-3TEV)-myc (Rad21*^{TEV}*)*	present study
w; P{w*$^{+}$*, tubpr<Rad21(271-3TEV)-myc*$_{10}$*<SV40}* (II.3), *P{w*$^{+}$*, tubpr<Rad21(271-3TEV)-myc*$_{10}$*<SV40}* (II.7)	*2x Rad21(271-3TEV)-myc (2x Rad21*^{TEV}*)*	present study
**Rad21-excision + transgenic Rad21 (+/- TEV-sites)**		
w; Rad21^{ex15}*, P{w*$^{+}$*, tubpr<Rad21(550-3TEV)-myc*$_{10}$*<SV40}* (III)	*Rad21*^{ex15}*, Rad21*^{TEV}	present study
w; Rad21*^{ex8}*, P{w*$^{+}$*, tubpr<Rad21(271-3TEV)-myc*$_{10}$*<SV40}* (III)	*Rad21*^{ex8}*, Rad21*^{TEV}	present study
w; P{w^{+}*, tubpr<Rad21(271-3TEV)-myc*$_{10}$*<SV40}* (II.3), *P{w*$^{+}$*, tubpr<Rad21(271-3TEV)-myc*$_{10}$*<SV40}* (II.7); *Rad21*^{ex3}	*2x Rad21*^{TEV}*; Rad21*^{ex3}	present study
w; Rad21^{ex3}*, P{w*$^{+}$*, tubpr-Rad21-myc*$_{10}$*-SV40}* (III)	*Rad21*^{ex3}*, Rad21*	present study
w; Rad21^{ex15}*, P{w*$^{+}$*, tubpr<Rad21(550-3TEV)-myc*$_{10}$*<SV40}, P{w*$^{+}$*, mhc-Gal80}* (III)	*Rad21*^{ex15}*, Rad21*^{TEV}*, mhc-Gal80*	present study
**Rad21-excision + Gal4 (+ TEV-protease or transgenic Rad21**^{TEV}**)**		
w; Rad21^{ex3}*, hs-Gal4/TM3, Sb, Kr-Gal4, UAS-GFP*	*Rad21*^{ex3}*, hs-Gal4/TM3, Kr-Gal4, UAS-GFP*	present study
w; P{w*$^{+}$*, α4-tub-Gal4-VP16}/CyO, wg-lacZ; Rad21*^{ex3}*/TM3, Sb, ubx-lacZ*	*α4-tub-Gal4/CyO, wg-lacZ; Rad21*^{ex3}*/TM3, ubx-lacZ*	present study
w; P{w*$^{+}$*, α4-tub-Gal4-VP16}, P{w*$^{+}$*, His2Av-mRFP1}* (II.2), *P{w*$^{+}$*, EGFP-Cid}* (II.1)*/CyO, wg-lacZ; Rad21*^{ex3}*/TM3, Sb, ubx-lacZ*	*α4-tub-Gal4, His2Av-mRFP1, EGFP-Cid/CyO; Rad21*^{ex3}*/TM3, ubx-lacZ*	present study

w; P{w+, GawB}F4/CyO, Kr-Gal4, UAS-GFP; Rad21^{ex15}, P{w^{+}, tubpr<Rad21(550-3TEV)-myc$_{10}$<SV40}*	*F4-Gal4/CyO, Kr-Gal4, UAS-GFP; Rad21^{ex15}, Rad21(550-3TEV)-myc*	present study
w; Rad21^{ex3}, P{w^{+}, UAS-NLS-v5-TEV-NLS$_{2}$}, elav-Gal4/TM3, Sb, Kr-Gal4, UAS-GFP	*Rad21^{ex3}, UAS-TEV, elav-Gal/TM3, Kr-Gal4, UAS-GFP*	present study
w; nsyb-Gal4/CyO; Rad21^{ex3}, P{w^{+}, UAS-NLS-v5-TEV-NLS$_{2}$}/TM3, Sb, Kr-Gal4, UAS-GFP	*nsyb-Gal4/CyO; Rad21^{ex3}, UAS-TEV/TM3, Kr-Gal4, UAS-GFP*	present study
w; Rad21^{ex3}, P{w^{+}, UAS-NLS-v5-TEV-NLS$_{2}$}, H24-Gal4, UAS-mCD8-GFP/TM6B, Tb	*Rad21^{ex3}, UAS-TEV, H24-Gal4, UAS-mCD8-GFP/TM6B, Tb*	present study
w; 201Y-Gal4, UAS-mCD8-GFP/(CyO); Rad21^{ex15}, P{w^{+}, tubpr<Rad21(550-3TEV)-myc$_{10}$<SV40}	*201Y-Gal4, UAS-mCD8-GFP; Rad21^{ex15}, Rad21^{TEV}*	present study
w; 201Y-Gal4, UAS-mCD8-GFP/ (CyO); Rad21^{ex3}, P{w^{+}, tubpr-Rad21-myc$_{10}$-SV40}	*201Y-Gal4, UAS-mCD8-GFP; Rad21^{ex3}, Rad21*	present study
w; 201Y-Gal4, UAS-mCD8-GFP/CyO; Rad21^{ex3}, P{w^{+}, mhc-Gal80}, P{w^{+}, UAS-NLS-v5-TEV-NLS$_{2}$}/TM6B, Tb	*201Y-Gal4, UAS-mCD8-GFP/CyO; Rad21^{ex3}, mhc-Gal80, UAS-TEV/TM6B, Tb*	present study
w; Cha-Gal4; Rad21^{ex3}, P{w^{+}, UAS-NLS-v5-TEV-NLS$_{2}$}/TM6B, Tb	*Cha-Gal4; Rad21^{ex3}, UAS-TEV/TM6B, Tb*	present study
w, UAS-CD8-GFP; If/CyO; Rad21^{ex15}, P{w^{+}, tubpr<Rad21(550-3TEV)-myc$_{10}$<SV40}	*UAS-CD8-GFP; If/CyO; Rad21^{ex15}, Rad21^{TEV}*	present study
w; Rad21^{ex3}, P{w^{+}, tubpr-Rad21-myc$_{10}$-SV40}, P{w^{+}, UAS-NLS-v5-TEV-NLS$_{2}$}/TM6B, Tb*	*Rad21^{ex3}, Rad21, UAS-TEV/TM6B, Tb*	present study

Table S2: Rescue of *Rad21* excision alleles by ectopic expression of either Rad21-myc or Rad21^{TEV}-myc

	Relative Viability *			
	♂ ***Rad21^{ex16}/TM3 Sb, Kr>GFP***		♂ ***Rad21^{ex3}/TM3 Sb, Kr>GFP***	
	**Pupae$^{\#}$**	**Adults$^{\#\#}$**	**Pupae$^{\#}$**	**Adults$^{\#\#}$**
♀ ***Rad21^{ex16}/TM3 Sb, Kr>GFP***	0	0	0	0
♀ ***Rad21^{ex3}/TM3 Sb, Kr>GFP***	0	0	0	0
♀ ***Rad21^{ex3}, Rad21-myc***	95.1	101	105	93.9
♀ ***Rad21^{ex15}, Rad21(550-3TEV)-myc***	111	81.4	107	78.4
♀ ***Rad21^{ex8}, Rad21(271-3TEV)-myc***	87.5	96.6	90.6	96.4

* Relative Viability: percentage of rescued pupae/adults, normalized to the values obtained when *Rad21^{ex}/TM3* ***Sb, Kr>GFP*** males were crossed to *w^{1118}* females.

\# Rescued pupae were identified by the absence of GFP-expression ($n \geq 400$).

\#\# Rescued adults were identified by the absence of *Sb* ($n \geq 250$).

Supplemental Figures

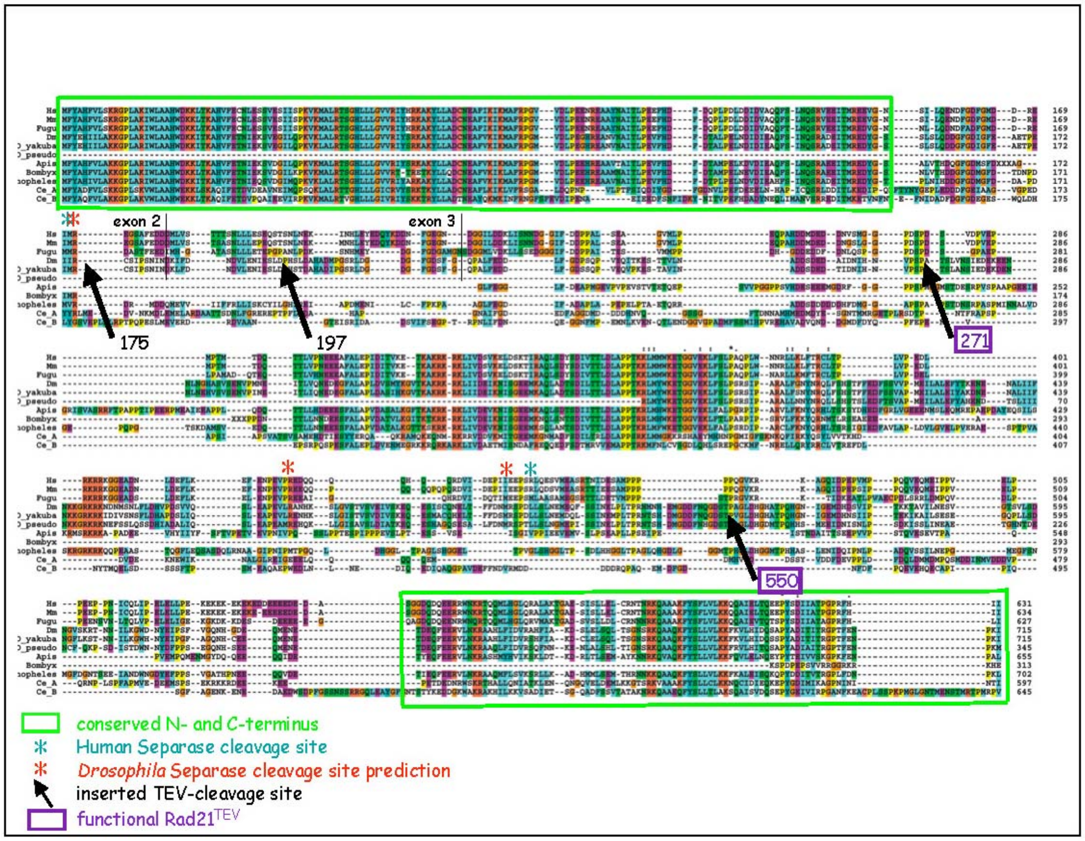

Figure S1. Multiple sequence alignment of protein-sequences of metazoan Rad21-homologs.

Annotated Rad21-protein sequences or sequence fragments were aligned to each other using ClustalW (Alexander Schleiffer, unpublished data). The conserved N-and C-terminal domains (green frames), human and (predicted) *D. melanogaster* separase cleavage sites (blue and red asterisks, respectively) and the boundaries of exons 2-3 and 3-4 are indicated. Four poorly conserved regions were chosen to introduce 3 tandem arrays of TEV recognition sequences (black arrows). The amino acid position after which the TEV sites had been introduced is indicated. TEV sites that rendered a functional Rad21^{TEV} protein (271 and 550) are highlighted in purple boxes.

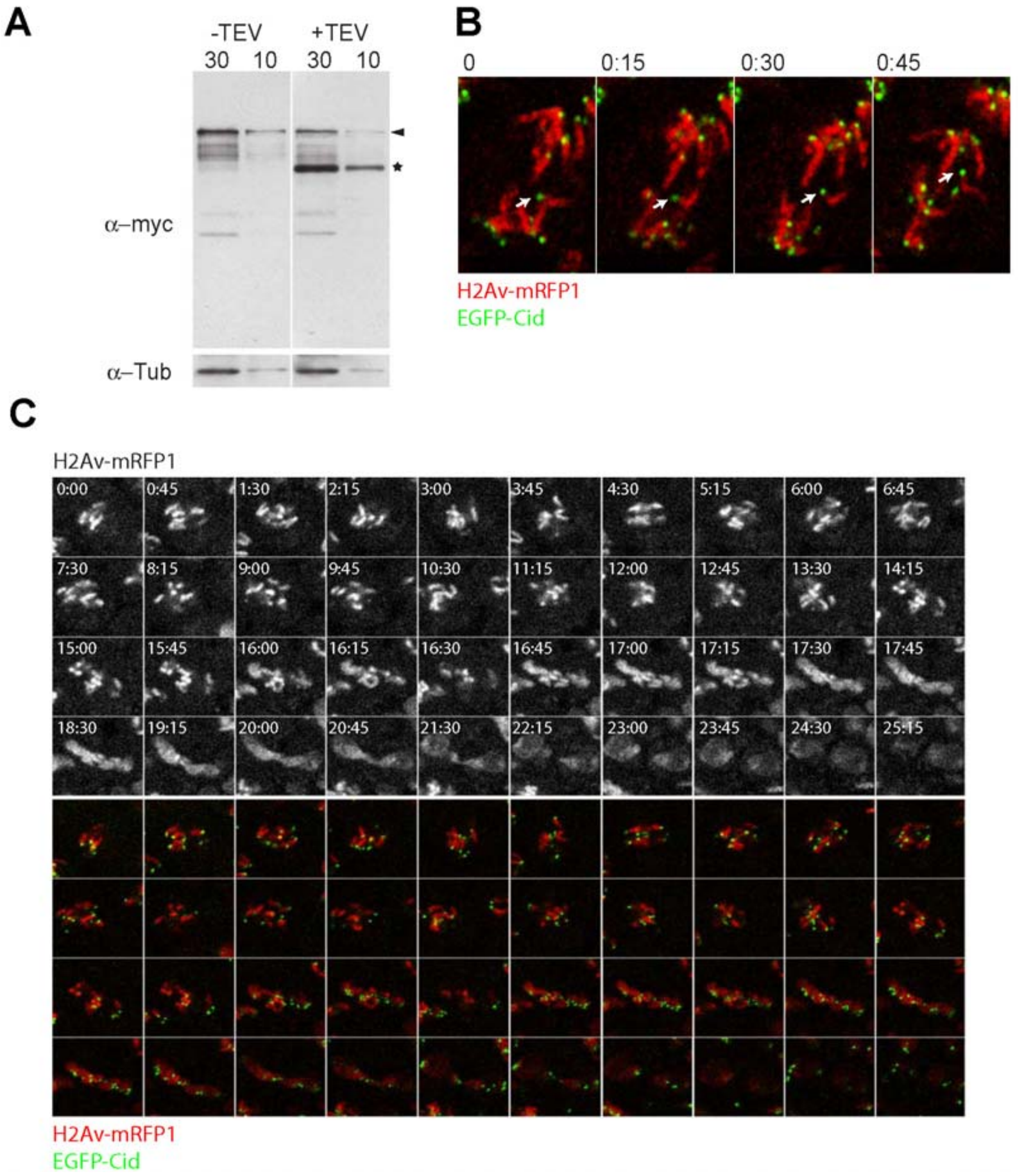

Figure S2. Rad21^{TEV} is cleaved by zygotically expressed TEV before mitosis 14.

(A) Total extracts corresponding to 30 or 10 embryos were analyzed by immunoblotting with antibodies against myc and α-tubulin 3-6 hours after egg deposition. (-TEV) Embryos surviving on myc-tagged Rad21^{TEV} express maternal Gal4, but do not contain the UAS-TEV transgene. (+TEV) Embryos surviving on myc-tagged Rad21^{TEV} express maternal Gal4, which drives zygotic TEV

expression. The arrowhead indicates the position of full-length myc-tagged $Rad21^{TEV}$, the asterisk that of the C-terminal TEV-cleavage product. Note that only 50% of the embryos used for the +TEV extracts contain the UAS-TEV construct (for details on genetic crosses see Supplemental Experimental Procedures). Therefore, the data is fully consistent with complete or almost complete cleavage of $Rad21^{TEV}$.

(B) Unstable kinetochore attachment after TEV expression. Frames shown were taken at 15 second intervals from a cell during the mitotic arrest resulting from zygotic TEV expression in embryos surviving on $Rad21^{TEV}$. DNA is marked with His2Av-mRFP1 (red), kinetochores with EGFP-Cid (green). The arrow follows the movement of a single kinetochore.

(C) Frames were taken at times indicated (min:sec) from a cell in a TEV-expressing embryo surviving on $Rad21^{TEV}$. DNA is marked with His2Av-mRFP1 (red), kinetochores with EGFP-Cid (green). After an initial mitotic arrest, chromosome decondensation starts abruptly (16:00) and unattached chromatids in the central region are cut by the cleavage furrow.

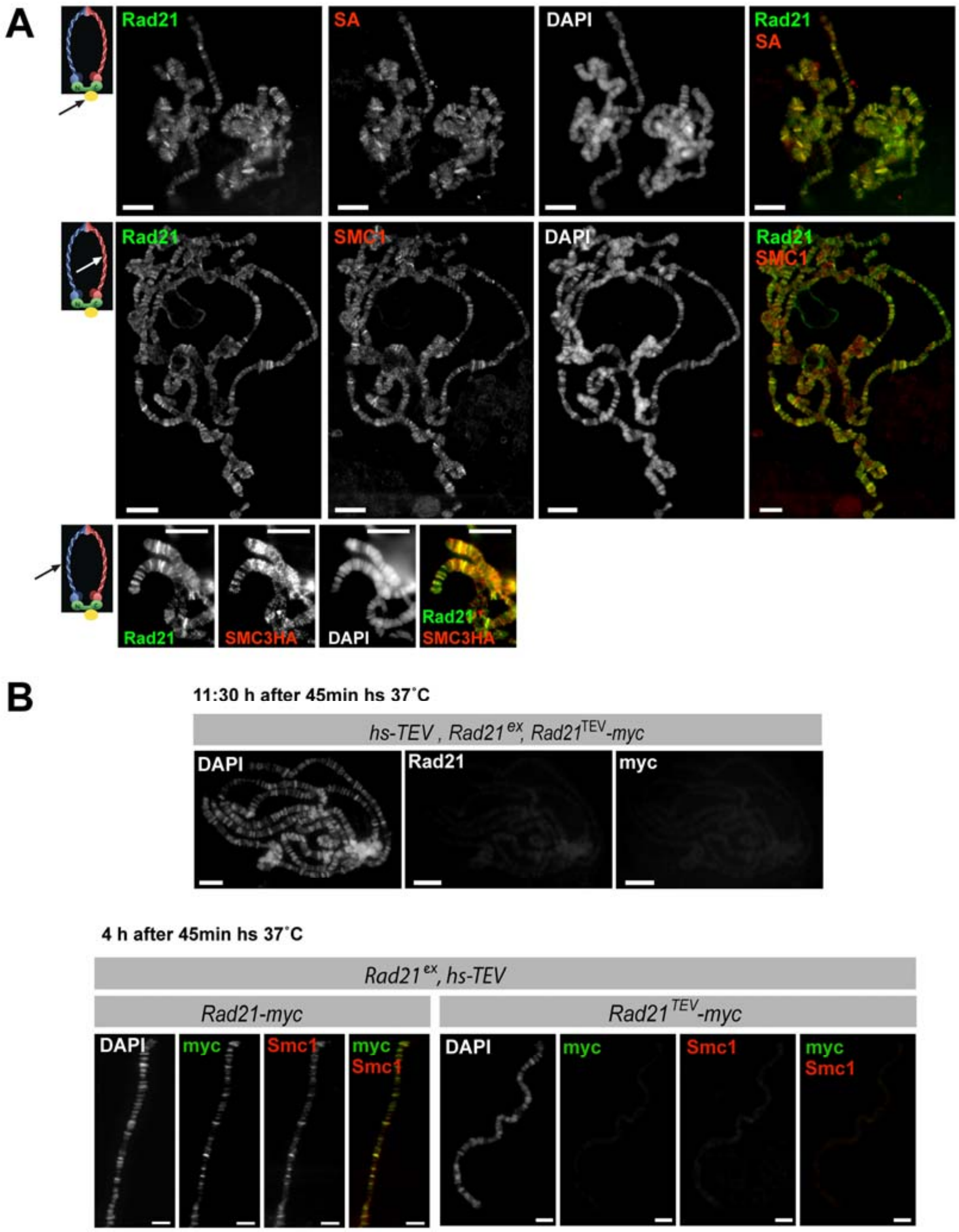

Figure S3. The cohesin complex binds to polytene chromosomes.

(A) Polytene chromosomes from wild-type flies (top two rows) or flies transgenic for HA-tagged SMC3 (bottom row) were coimmunostained with antibodies against endogenous Rad21 (green) and either endogenous SA (top row), endogenous SMC1 (middle row) or the HA-epitope (bottom row) (red). A schematic of the cohesin complex is shown at the left of each row (arrow points to the subunit costained with

Rad21). DNA was visualized with DAPI. Scale bars, 15µm.

(B) Polytene chromosome spreads from 3rd instar larvae, which express heat-inducible TEV (hs-TEV) and myc-tagged $Rad21^{TEV}$ as their only source of Rad21, were prepared after a 45 min heat shock. Spreads were coimmunostained with antibodies against endogenous Rad21 and myc (top) or myc (green) and SMC1 (red) (bottom). Note the absence of Rad21-, myc- and SMC1-staining after TEV cleavage of $Rad21^{TEV}$. DNA was visualized with DAPI. Scale bar, 15µm (top), 10µm (bottom).

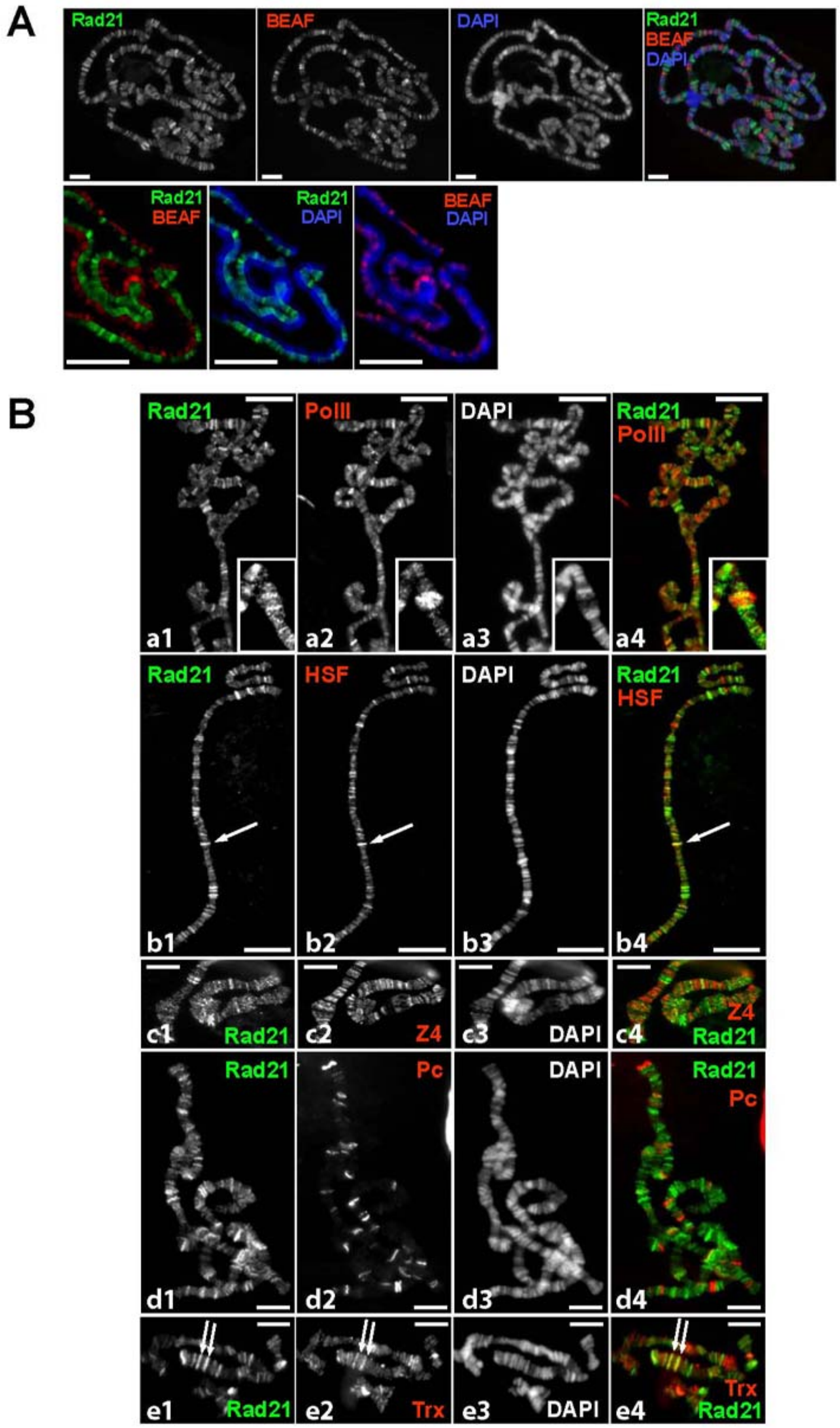

Figure S4. Rad21 binds to distinct regions on polytene chromosomes.

(A) Polytene chromosomes from wild-type flies (w^{1118}) were coimmunostained with

antibodies against Rad21 (green) and BEAF (red), a well-characterized boundary-associated factor. DNA is shown in blue in merged images. Two different magnifications are shown (top row: 40x objective; bottom row: 100x objective, split channels). BEAF and Rad21 localize to distinct interband regions. Scale bar, 20µm. (B) Polytene chromosomes from wild-type flies (w^{1118}) were coimmunostained with antibodies against Rad21 and (a) PolII (RNA Polymerase II), (b) HSF (Heat-Shock Factor), (c) Z4 (interband-specific Zinc-finger protein), (d) Pc (Polycomb) and (e) trx (Trithorax). DNA was visualized with DAPI. As seen in the merged images (Rad21 in green, other proteins in red), the distribution of cohesin and of the other tested factors differs significantly. White arrows point to the few regions, in which an overlap between Rad21 and the other factor tested could be detected. The insets in a1-a4 show a higher magnification (3x) of a PolII-stained chromosomal puff, which is flanked by Rad21-bound regions. Scale bars, 20µm (a and b), 10µm (c-e).

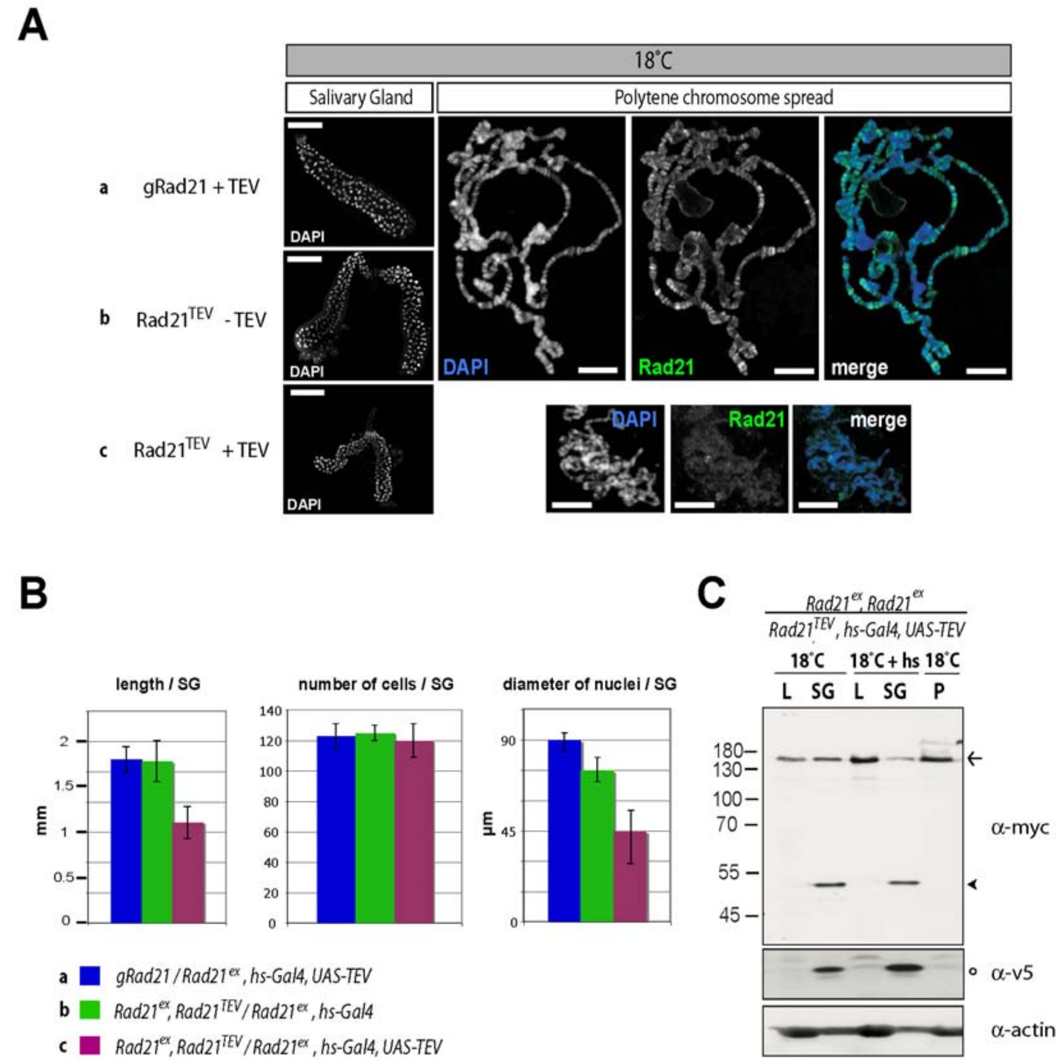

Figure S5. Cohesin is required for salivary gland development.

(A) Salivary glands were dissected from 3rd instar larvae that had been raised at 18˚C throughout development. (Left) In the whole-mount preparations, DNA was stained with DAPI. (Right) Polytene chromosome spreads were immunostained with antibodies against Rad21 (green). DNA was visualized with DAPI (blue). Three different genotypes were compared: a) *gRad21/Rad21*^{ex3}, *hs-Gal4, UAS-TEV*; b) *Rad21*^{ex15}, *Rad21*^{TEV}*/Rad21*^{ex3}, *hs-Gal4*; c) *Rad21*^{ex15}, *Rad21*^{TEV}*/Rad21*^{ex3}, *hs-Gal4, UAS-TEV*. Since polytene chromosome spreads from genotypes (a) and (b) are similar, only a representative spread from (a) is shown. Note that salivary glands with reduced amounts of Rad21 have smaller but not fewer cells. Scale bar, 500µm (whole

mount salivary glands), 20μm (polytene chromosome spreads).

(B) Quantitative analysis of salivary glands average length (in mm), total number of cells per salivary gland and the average diameter per nucleus (in μm) was performed from salivary glands from 3rd instar larvae of the indicated genotypes (>10 per genotype). Larvae in which Rad21^{TEV} has been cleaved show smaller salivary glands with smaller nuclei. The number of cells per salivary glands remains unaltered.

(C) Protein extracts from a strain carrying hs-Gal4, UAS-TEV and surviving on myc-tagged Rad21^{TEV} were analyzed by Western Blotting. Extracts of larvae lacking salivary glands (L), dissected salivary glands (SG) or pupae (P) were prepared from crosses raised at 18°C. Samples from lanes 3 and 4 were prepared 1 hour after heat shock treatment (45 min 37°C). Western Blot analysis was performed with antibodies against myc (detecting full-length Rad21^{TEV}-myc (arrow) and the C-terminal TEV-cleavage fragment (arrowhead)), v5 (detecting TEV-protease) and actin (loading control). Before heat shock induction of TEV, significant levels of the protease could be detected in salivary glands (open circle). The TEV cleavage fragment of Rad21 is also observed. Neither TEV protease nor Rad21^{TEV} cleavage fragments were detected in larvae without salivary glands or pupae before TEV induction. A Molecular Weight Marker (in kDa) is shown on the left.

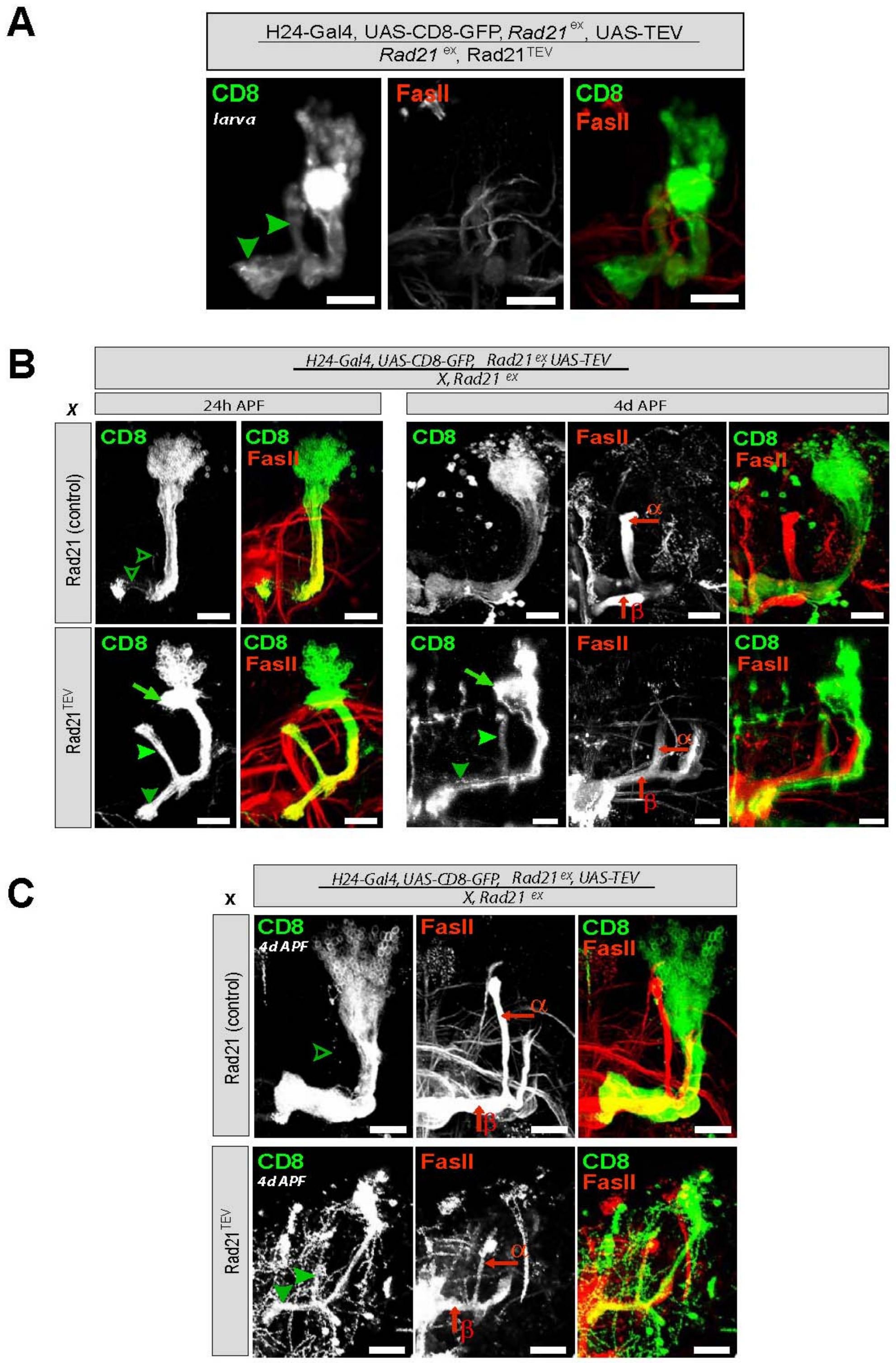

Figure S6. Analysis of pruning in γ neurons with H24-Gal4-induced TEV cleavage of Rad21.

H24-Gal4 was used to drive expression of TEV and mCD8 in γ neurons of the

mushroom body from flies that survived on transgenic Rad21 with ($Rad21^{TEV}$) or without (Rad21) TEV cleavage sites. Shown are maximal Z projections of single confocal sections of a right brain hemisphere, stained with antibodies against mCD8 (green) and FasII (red). Scale bars, 20µm.

(A) Larval γ neurons from a $Rad21^{TEV}$ brain project into the dorsal and medial lobes (filled green arrowheads).

(B) (24h APF, left) In the presence of Rad21 (control), γ neurons have pruned their medial and dorsal axon projections (open arrowheads) as well as their dendrites. In γ neurons of $Rad21^{TEV}$ pupae, larval axon-projections persist in the medial and dorsal lobes (filled green arrowheads). Note also the presence of unpruned dendrites (green arrow). (4d APF, right) In the presence of Rad21 (control), γ neurons have re-extended their axons medially towards the midline. Axons of α/β neurons in the dorsal and medial lobes are labeled with FasII (red arrows). In γ neurons of $Rad21^{TEV}$ pupae, larval axon-projections as well as dendrites (filled green arrowheads and green arrow, respectively) persist in the dorsal and medial lobe. Projections of α/β neurons are normal (red arrows).

(C) In the presence of Rad21 (top), γ neurons are tightly bundled and project exclusively towards the midline at 4d APF. No γ neurons are found in the FasII-positive dorsal lobe (open green arrowhead). In the absence of Rad21 (bottom), γ neuronal projections persist in the dorsal and medial lobes (filled green arrowheads), but are often disorganized and mistargeted. Although the FasII staining for α/β neurons appears weaker than in the control, the projection pattern is normal.

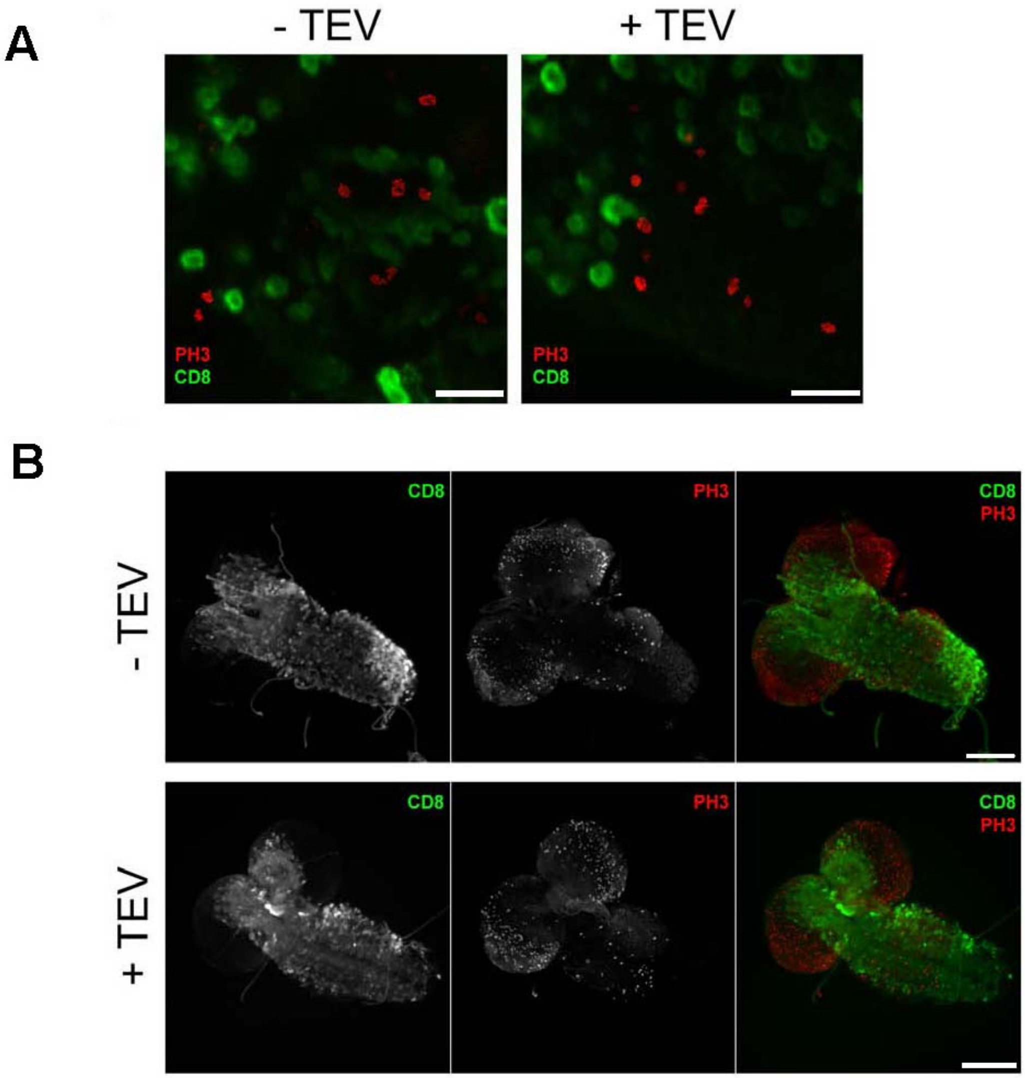

Figure S7. TEV cleavage of Rad21 in cholinergic-neurons does not cause mitotic defects.

Brains from control (-TEV: *UAS-CD8-GFP; Cha-Gal4/CyO; Rad21*^{ex15}, *Rad21*^{TEV}*/TM6B*) and TEV-cleaved brains (+TEV: *UAS-CD8-GFP; Cha-Gal4/CyO; Rad21*^{ex15}, *Rad21*^{TEV}*/Rad21*^{ex3}, *UAS-TEV*) were immunostained for CD8 (green) and the mitotic marker phospho-histone H3 (PH3, red).

A) High magnification images show that there is no co-localization between the CD8 positive cells and the mitotic marker. Additionally, mitotic figures in TEV-cleaved brains look similar to the controls. Scale bars, 20µm.

B) Confocal images of brains and ventral nerve cords from both control and TEV-cleaved brains. PH3 staining reveals that there is no detectable accumulation of mitotic figures after TEV cleavage, and that there are no obvious morphological defects in the CD8 positive cholinergic neurons. Scale bars, 100µm.
